# Neferine Ameliorates Severe Acute Pancreatitis-Associated Intestinal Injury by Promoting NRF2-mediated Ferroptosis

**DOI:** 10.7150/ijbs.112888

**Published:** 2025-04-28

**Authors:** Lilong Zhang, Kunpeng Wang, Wanrong Jiang, Pingan Ding, Weixing Wang, Kailiang Zhao, Chen Chen

**Affiliations:** 1 Department of General Surgery, Renmin Hospital of Wuhan University, Wuhan, 430060, Hubei, China.; 2 Hubei Key Laboratory of Digestive System Disease, Renmin Hospital of Wuhan University, Wuhan, 430060, Hubei, China.; 3 General Surgery Laboratory, Renmin Hospital of Wuhan University, Wuhan, 430060, Hubei, China.; 4 Central laboratory, Renmin Hospital of Wuhan University, Wuhan, 430060, Hubei, China.; 5 The Third Department of Surgery, the Fourth Hospital of Hebei Medical University, Shijiazhuang, 050011, Hebei, China.

**Keywords:** Neferine, Acute pancreatitis, Intestinal injury, Gut microbiota, Iron export, Ferroptosis

## Abstract

Severe acute pancreatitis (SAP) is a life-threatening abdominal condition often complicated by intestinal barrier dysfunction, which further exacerbates disease progression. Neferine has demonstrated potent anti-inflammatory and antioxidant properties; however, its role in ameliorating SAP and associated intestinal barrier damage remains unclear. In this study, we found that neferine administration significantly alleviates SAP severity by reducing pancreatic and ileal pathological damage, oxidative stress, inflammatory cell infiltration, and intestinal flora translocation. Additionally, neferine enhances the expression of tight junction proteins, increases short-chain fatty acid levels, and improves intestinal dysbiosis, thereby contributing to intestinal homeostasis restoration. Mechanistically, neferine upregulates Nrf2 expression and promotes its nuclear translocation by competitively binding to the Cys-288 site on Keap1. This activation enhances the Nrf2/FPN and Nrf2/xCT/GPX4 axes, thereby preventing ferroptosis and ultimately protecting against pancreatic and intestinal injury in SAP mice. Furthermore, the protective effects of neferine were largely reversed by the Nrf2 inhibitor ML385 and the ferroptosis inducer erastin. This study demonstrates that neferine effectively alleviates SAP by inhibiting ferroptosis and restoring intestinal homeostasis, providing insights into new treatment options for SAP.

## Introduction

Acute pancreatitis (AP) is a common and urgent abdominal condition with complex pathophysiology, leading to autodigestion of pancreatic parenchyma and excessive production of pro-inflammatory cytokines 1. Severe acute pancreatitis (SAP) can precipitate systemic inflammatory response syndrome (SIRS) and multiple organ dysfunction syndrome (MODS), with mortality rates ranging from 36% to 50% 2, 3. Due to their anatomical proximity and direct connection to the pancreas, the intestines are particularly vulnerable to inflammation and severe damage during pancreatitis onset 4. Previous studies have shown that the intestine is the most affected organ during AP 5. Current treatment strategies for AP and its associated intestinal injuries remain suboptimal, highlighting the urgent need for more precise and effective therapeutic options.

Ferroptosis is a recently identified form of cell death 6, characterized by inhibited glutathione (GSH)-GPX4 activity, which leads to the accumulation of reactive oxygen species (ROS). This, in turn, triggers excessive lipid peroxidation, including the production of MDA, which disrupts cell membrane integrity and induces ferroptosis 7, 8. The role of ferroptosis in AP is increasingly recognized. Research suggests that inhibiting ferroptosis reduces ROS and inflammation in AP while also mitigating organ dysfunction, including acute kidney injury and acute lung injury, associated with SAP 9.

The green embryo of the *Nelumbo nucifera* Gaertn seed, referred to as Nelumbinis plumula, is a widely utilized edible Chinese herb, commonly brewed as tea in China 10. This herb is notable for being both abundant and cost-efficient while offering diverse nutritional and therapeutic benefits, including positive effects on cardiovascular health, neurological conditions, and anxiety 11. Nelumbinis plumula contains various bioactive compounds, such as flavonoids, volatile oils, alkaloids, and glycosides, with alkaloids representing its dominant components 12. Among these, neferine is the most prevalent alkaloid and one of the three key bisbenzylisoquinoline alkaloids. Neferine demonstrates favorable bioavailability and pharmacological potential. There is substantial evidence indicating that neferine exhibits anti-inflammatory and antioxidant properties 13, as well as anti-pulmonary fibrosis activity 14. Although the protective effects of neferine in a variety of diseases are well established, its therapeutic role in AP has not been studied. Additionally, studies suggest that neferine is characterized by low toxicity and negligible side effects, with no observed adverse effects on the liver or kidneys in diabetic mice 14. These findings underscore the safety of neferine and its promising potential for future development and clinical applications.

Li *et al.* 15 demonstrated that neferine induces ferroptosis in thyroid cancer by inhibiting the Nrf2/HO-1 pathway, and this is the only report exploring the relationship between neferine and ferroptosis. However, our research uncovered a very interesting fact. Instead of promoting ferroptosis, neferine upregulates Nrf2 expression and facilitates its nuclear translocation by competitively binding to the Cys-288 site on Keap1 in SAP mice. This prevents ferroptosis, ultimately protecting against pancreatic and intestinal injury in SAP mice. Moreover, neferine exerts protective effects by modulating the gut microbiota and promoting the synthesis of short-chain fatty acids (SCFAs).

## Materials and Methods

### Animals

All procedures adhered to the ARRIVE guidelines and ethical standards set by the National Institutes of Health 16. Approval for the protocols was obtained from the animal ethics committee of our hospital (Approval No. WDRM20231202A).

### Induction of SAP and drug treatments

The mice were randomly assigned to one of five groups (n = 6): the control (CON) group, the neferine (NE) group, the caerulein plus LPS (SAP) group, the low neferine + caerulein plus LPS (Low-NE+SAP) group, the high neferine + caerulein plus LPS (High-NE+SAP or NE+SAP) group. Lipopolysaccharide (LPS) was obtained from Sigma-Aldrich (St. Louis, Missouri, USA); ML385, Fer-1, and erastin were sourced from MedChemExpress (Shanghai, China); caerulein and neferine were obtained from Yuanye Bio-Technology (Shanghai, China). The SAP model was established as previously described in the literature 17. Mice received ten intraperitoneal injections of 100 μg/kg caerulein, spaced one hour apart. After the final caerulein injection, LPS (10 mg/kg) was administered intraperitoneally.

Prior to SAP induction, neferine was dissolved in a solvent (10% DMSO, 40% PEG300, 5% Tween-80, 45% saline) and administered intraperitoneally at 75 mg/kg/day (high-NE) or 50 mg/kg/day (low-NE) for one week 18. At 24 hours post-caerulein injection, the mice were anesthetized with chloral hydrate and euthanized. Blood, distal ileum, pancreatic tissue, and cecal lumen contents were harvested for analysis.

ML385 (30 mg/kg) was administered intraperitoneally 1 hour before caerulein treatment to inhibit Nrf2 activity, as reported previously 19. Fer-1 (5 mg/kg) or erastin (30 mg/kg) was also injected 1 hour prior to caerulein 19, 20. Fer-1 prevents phospholipid peroxidation and cellular ferroptosis by reducing membrane lipid damage, while erastin induces ferroptosis by inhibiting GSH synthesis and GPX4 activity.

### Cell culture and plasmid transfection

The 293T and IEC-6 cell lines were cultured at 37 °C in a 5% CO_2_ atmosphere in DMEM (Servicebio, Wuhan, China), supplemented with 1% antibiotics (Biosharp, Hefei, China) and 10% fetal bovine serum (QmSuero, Wuhan, China). AR42J cells was cultured in DMEM/F12 (Servicebio, Wuhan, China) with 20% fetal bovine serum. Additionally, IEC-6 culture required 10 µg/mL of insulin. Lentivirus was produced by transfecting 293T cells with either the si-Nrf2 or si-FPN plasmid, along with psPAX2 and pMD2G (DesignGene, Wuhan, China), using Lipo8000 (Beyotime, China). After 48 hours, the virus-containing supernatant was collected, mixed with serum and polycoagulant (Biosharp, Hefei, China), and introduced into exponentially growing AR42J and IEC-6 cells. Stable knockdown of Nrf2 (si-Nrf2) or FPN (si-FPN) was achieved by selecting cells with puromycin (Biosharp, Hefei, China) for two days. Wild-type and Cys-288 mutant Keap1 plasmids were obtained from MIAOLING BIOLOGY (Wuhan, China). IEC-6 and AR42J were transfected using Lipo8000 reagent (Beyotime, China) according to the manufacturer's instructions. To simulate pancreatic acinar cell and intestinal epithelial cell damage during pancreatitis, IEC-6 and AR42J were exposed to TNF-α (50 ng/mL) for 24 hours 21.

For detailed methodological information, please refer to the supplementary methods.

## Results

### Neferine dose selection

We initially evaluated the safety profile of various doses of neferine. The results showed no significant differences in body weight among the high-neferine-treated, low-neferine-treated, and control mice (Fig. S1A). Additionally, we observed no significant changes in biochemical markers, including ALT, AST, creatinine, and urea levels across all three groups (Fig. S1B, S1C). H&E staining did not reveal any notable morphological or pathological changes in the liver, lungs, heart, and kidneys (Fig. S1D). Collectively, these findings suggest that neferine exhibits minimal toxicity.

**Figure 1:**
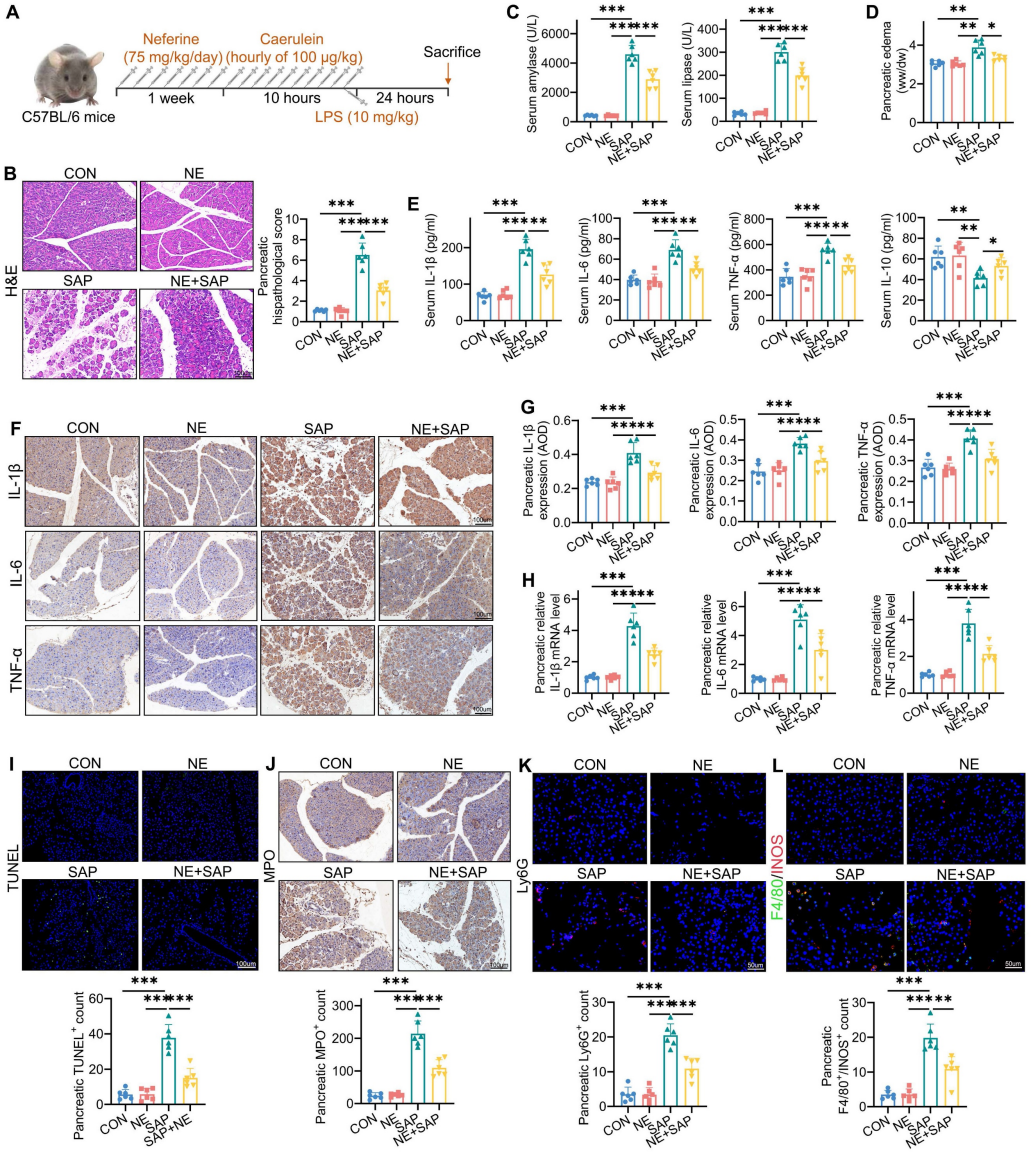
Neferine mitigates the severity of SAP. (A) Experimental schedule for SAP establishment. (B) H&E staining of pancreatic tissue and corresponding histologic scores. (C) Serum levels of amylase and lipase. (D) Pancreatic edema assessed by the wet-to-dry ratio. (E) Serum levels of IL-1β, IL-6, TNF-α, and IL-10. (F, G) Immunohistochemical staining of pancreatic IL-1β, IL-6, and TNF-α (F), along with average optical density calculations (G). (H) Pancreatic mRNA levels of* il-1β*, *il-6*, and *tnf-α*. (I) Immunofluorescence staining of pancreatic TUNEL and quantification of TUNEL^+^ cells. (J) Immunohistochemical staining of pancreatic MPO and quantification of MPO^+^ cells. (K) Immunofluorescence staining of pancreatic Ly-6G and quantification of Ly6G⁺ neutrophils. (L) Immunofluorescence staining of F4/80 and INOS in the pancreas, with quantification of F4/80^+^ iNOS^+^ macrophages. AOD, average optical density; NE, Neferine; Data were expressed as mean ± SD; n = 6 in each group. *P < 0.05, **P < 0.01, ***P < 0.001.

To assess the therapeutic effects of neferine, we first evaluated its impact on the caerulein plus LPS-induced SAP model, as outlined in Fig. 1A. Histological analysis demonstrated that treatment with neferine significantly reduced pancreatic injury in the SAP mice (Fig. 1B, S1E). Furthermore, we found that high-dose neferine treatment was significantly more effective than low-dose neferine (Fig. S1E). Based on these results, high-dose neferine was selected for all subsequent studies.

### Neferine alleviates the severity of SAP

SAP mice exhibited pronounced pancreatic edema and elevated serum amylase/lipase levels compared to both the control and neferine-only treatment groups (Fig. 1C, 1D). Notably, neferine administration significantly attenuated pancreatic edema and enzyme levels in SAP-challenged mice. To further characterize the inflammatory cascade in SAP pathogenesis, we quantified key cytokines (IL-1β, IL-6, TNF-α, and IL-10) using triangulated analysis across pancreatic tissue and serum. SAP induction triggered a robust upregulation of pro-inflammatory mediators (IL-1β, IL-6, TNF-α), while neferine treatment demonstrated potent anti-inflammatory effects, significantly reducing both systemic and tissue-localized cytokine storms (Fig. 1E-H). Additionally, TUNEL staining revealed increased pancreatic apoptosis in SAP mice, which was reduced in neferine-treated SAP mice (Fig. 1I).

MPO activity, a marker of inflammatory cell infiltration, is secreted by neutrophils in inflamed tissues 22. We observed that MPO levels were significantly lower in neferine-treated SAP mice compared to SAP mice (Fig. 1J). To further assess neutrophil infiltration, we performed Ly6G staining, which confirmed that neferine treatment substantially reduced neutrophil presence (Fig. 1K). During the progression of AP, macrophages transition from an M1 to an M2 phenotype. M1 macrophages dominate during the acute phase, while M2 macrophages increase during tissue repair and regeneration 23. We evaluated the infiltration of M2 macrophages (F4/80^+^CD206^+^) and M1 macrophages (F4/80^+^iNOS^+^) in the pancreas. The results revealed that neferine treatment significantly reduced the infiltration of M1 macrophages and increased the presence of M2 macrophages in SAP mice (Fig. 1L, S2). In summary, these findings demonstrate that neferine exerts anti-inflammatory effects in SAP mice.

### Neferine protected against intestinal barrier dysfunction and reduced bacterial translocation

**Figure 2:**
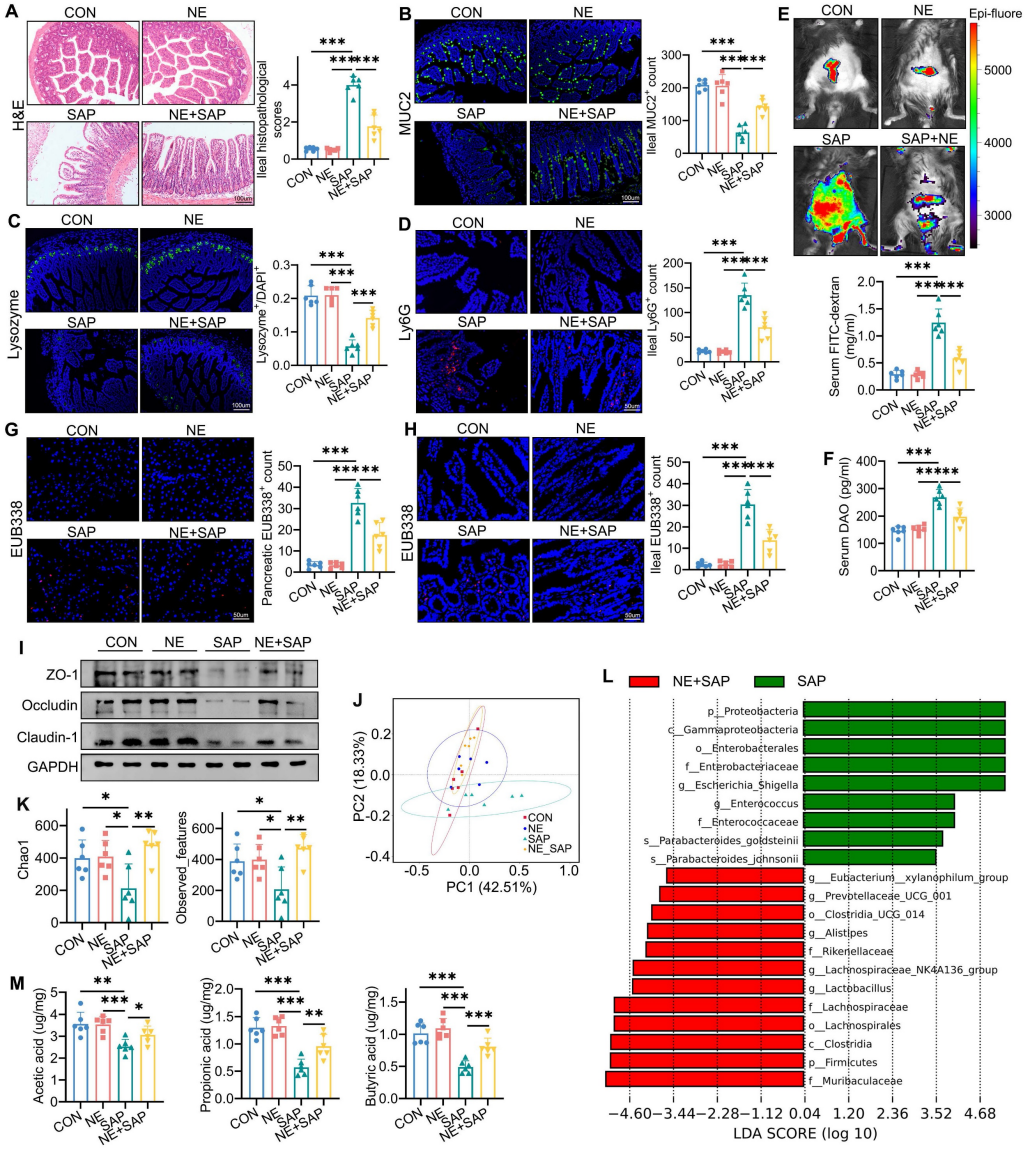
Neferine attenuates ileal damage in SAP. (A) Representative H&E staining of the ileum and corresponding histopathology scores. (B) Immunofluorescence staining of ileal MUC2 and its quantification. (C) Immunofluorescence staining of ileal lysozyme and its quantification. (D) Immunofluorescence staining of ileal Ly-6G and quantification of Ly6G⁺ neutrophils. (E) FITC-dextran distribution detected by small-animal imaging, and serum FITC-dextran levels measured. (F) Serum DAO levels measured by ELISA. (G, H) Detection of total bacterial hybridization signals using the EUB338 probe in the pancreas (G) and ileum (H), with quantification of EUB338-positive bacteria in the epithelium per field. (I) Western blot analysis of ZO-1, Occludin, and Claudin-1 protein levels in the ileum. (J) Distributional differences in gut microbiota profiles assessed using PCoA. (K) Community richness estimated by Chao1 and Observed_features. (L) Differentially abundant taxa between the SAP and GAA/SAP groups identified by linear discriminant analysis effect size (LEfSe) analysis. (M) Fecal levels of acetic acid, propionic acid, and butyric acid. NE, Neferine; Data are presented as mean ± SD; n = 6 per group. *P < 0.05, **P < 0.01, ***P < 0.001.

Intestinal dysfunction, characterized by inflammation and structural damage, is a major complication of SAP and exacerbates its progression. To determine whether neferine treatment preserves intestinal homeostasis and mitigates SAP progression, we conducted histopathological assessments of ileal tissues. Neferine administration significantly alleviated SAP-induced ileal injury (Fig. 2A) and reduced intestinal pro-inflammatory cytokine levels, including IL-1β, IL-6, and TNF-α (Fig. S3A-D). TUNEL staining revealed heightened ileal cell apoptosis under SAP conditions, which was notably reversed by neferine treatment (Fig. S4A).

MUC2, secreted by goblet cells, is a key component of the intestinal mucus barrier and serves as a primary defense mechanism 24. Similarly, lysozyme, produced by Paneth cells, plays a critical role in intestinal homeostasis by preventing bacterial invasion, and its dysregulation is linked to compromised barrier function 25. Immunofluorescence (IF) analysis demonstrated partial recovery of MUC2 and lysozyme expression following neferine treatment (Fig. 2B, 2C). Neferine pretreatment also significantly reduced intestinal neutrophil (Ly6G^+^) infiltration in SAP mice (Fig. 2D).

Increased intestinal permeability facilitates the translocation of pathogenic bacteria and endotoxins to the pancreas, aggravating pancreatic injury and secondary septic infections in SAP 26. Serum diamine oxidase (DAO), an intracellular enzyme predominantly expressed in intestinal villous cells, serves as a serum biomarker of intestinal barrier integrity 27. As shown in Fig. 2E and 2F, the SAP challenge induced a significant increase in serum DAO and FITC levels, an effect that was ablated by neferine pretreatment. Small-animal imaging of FITC-dextran distribution revealed higher fluorescence intensity in the control and neferine groups compared to the SAP group, with intermediate levels observed in the NE/SAP group (Fig. 2E). Furthermore, EUB338 probe testing of ileal and pancreatic tissues demonstrated that neferine mitigated SAP-induced bacterial translocation (Fig. 2G, 2H). Notably, neferine restored the expression of ZO-1, occludin, and claudin-1, which were downregulated under SAP conditions (Fig. 2I, S4B). Collectively, our findings indicate that neferine treatment enhances intestinal barrier integrity and reduces bacterial translocation during SAP progression.

### Neferine restored intestinal microbiota abundance in SAP

Intestinal dysbiosis plays a critical role in the progression of SAP 28. To investigate changes in gut microbiota during SAP, we performed 16S rRNA sequencing. Fig. S5A shows the ASVs shared among the groups and those unique to each group, as illustrated in a Venn diagram. PCoA and NMDS analyses revealed significant differences in gut microbiota composition between the SAP group and the other three groups (Fig. 2J, S5B). Community richness, assessed using Chao1 and Observed_features indices, and diversity, measured by Shannon and Pielou_e indices, were significantly reduced in SAP mice compared to the control and neferine groups. However, neferine supplementation partially restored richness and diversity in SAP mice (Fig. 2K, S5C). Fig. S5D shows the taxonomic composition distribution at the genus level in fecal samples from the four groups. LEfSe analysis revealed that, compared to SAP mice, the intestinal microbiota of neferine-treated SAP mice was enriched in *Lactobacillus*, *Lachnospiraceae_NK4A136_group*, *Alistipes*, *Prevotellaceae_UCG-001*, and *Eubacterium_xylanophilum_group*, while *Escherichia-Shigella* and *Enterococcus* were reduced (Fig. 2L). The distribution of the above different bacteria genera in the four groups is further presented in Fig. S5E.

SCFAs produced by intestinal microbiota exhibit anti-inflammatory properties and play a crucial role in maintaining gut barrier integrity and function, thereby promoting gastrointestinal homeostasis 29. We found that neferine treatment reversed the SAP-induced reduction in SCFA levels, including acetic acid, propionic acid, butyric acid, isobutyric acid, valeric acid, and isovaleric acid (Fig. 2M, S5F). Thus, neferine can reshape intestinal microbiota and increase the production of SCFAs.

### Neferine attenuates SAP-induced ferroptosis

**Figure 3:**
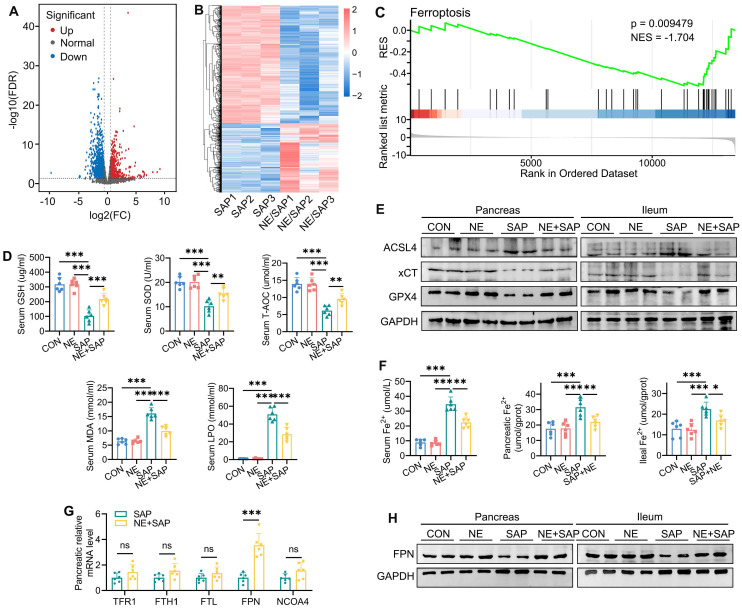
Neferine restores iron metabolism by regulating FPN-mediated iron export. (A) Volcano plot: gray, red, and blue dots represent genes with no significant difference, significantly upregulated genes, and significantly downregulated genes, respectively (fold change ≥ 1.5 and FDR < 0.05). (B) Heatmap of differentially expressed genes: each row represents a gene, and each column represents a sample, with red indicating upregulation and blue indicating downregulation. (C) Gene set enrichment analysis (GSEA) of the ferroptosis signaling pathway. (D) Serum levels of GSH, SOD, T-AOC, MDA, and LPO. (E) Western blot analysis of ACSL4, xCT, and GPX4 protein levels in the pancreas and ileum. (F) Serum, pancreatic, and ileal Fe²⁺ levels. (G) Relative quantification of *Tfr1*, *Fth1*, *Ftl*, *Fpn*, and *Ncoa4* mRNA levels in the pancreas. (H) Western blot of FPN protein levels in the pancreas and ileum. NE, Neferine; Data were expressed as mean ± SD; n = 6 in each group. *P < 0.05, **P < 0.01, ***P < 0.001.

To better understand how neferine alleviates pancreatic and intestinal damage in SAP mice, RNA sequencing was performed on pancreatic tissues. Analysis of differential gene expression revealed that 144 genes were significantly upregulated, while 357 were markedly downregulated in the pancreatic tissues of neferine-treated SAP mice compared to the SAP group (FDR < 0.05 and fold change ≥ 1.5) (Fig. 3A and 3B). GSEA further demonstrated that neferine treatment notably suppressed ferroptosis in SAP mice (Fig. 3C).

Ferroptosis is characterized by the buildup of redox-active iron, a reduction in GSH levels, and lipid peroxidation. To investigate this further, we measured serum levels of GSH, superoxide dismutase (SOD), total antioxidant capacity (T-AOC), malondialdehyde (MDA), and lipid peroxidation (LPO). The results revealed that SAP induced a downregulation of GSH, SOD, and T-AOC levels, along with an upregulation of MDA, and LPO, effects that were reversed by neferine treatment (Fig. 3D). We further detected changes in MDA, GSH, and SOD in pancreatic and intestinal tissues and found that the trend was consistent with the changes observed in serum (Fig. S6A, S6B). The expression of GPX4 and xCT (also known as SLC7A11), key biomarkers of ferroptosis that protect against lipid peroxidation, was reduced following SAP. Neferine administration reversed these SAP-induced alterations in GPX4 and xCT levels (Fig. 3E, S6C, S6D). ACSL4 facilitates the synthesis of lipids containing polyunsaturated fatty acids, thereby promoting ferroptosis 30. The results showed that SAP increased the expression of ACSL4, while neferine treatment alleviated this effect (Fig. 3E, S6C-E).

### Neferine restores iron metabolism via regulating FPN-mediated iron export

Iron plays a crucial role in ferroptosis by promoting lipid ROS production via the Fenton reaction 31, prompting us to examine whether neferine influences iron metabolism in the pancreas and ileum. SAP induced substantial free iron accumulation in tissues, as evidenced by elevated Fe²⁺ levels in serum, pancreas, and ileum, an effect that was reduced by neferine treatment (Fig. 3F).

To investigate how neferine-regulated iron metabolism, we analyzed the expression of key iron-regulating genes in the pancreas, including those involved in iron uptake (TFR1), export (FPN), storage (FTH1, FTL), and ferritinophagy (NCOA4). Notably, neferine selectively increased FPN mRNA levels in SAP mice without affecting the expression of other iron-related genes (Fig. 3G). Additionally, SAP significantly reduced FPN protein expression in the pancreas and ileum, an effect that was reversed by neferine (Fig. 3H, S6F). These results suggest that SAP disrupts iron homeostasis, while neferine restores balance by enhancing FPN-mediated iron export.

### Upregulation of ferroptosis reversed the protective effects of neferine on pancreatic and ileal injury

**Figure 4:**
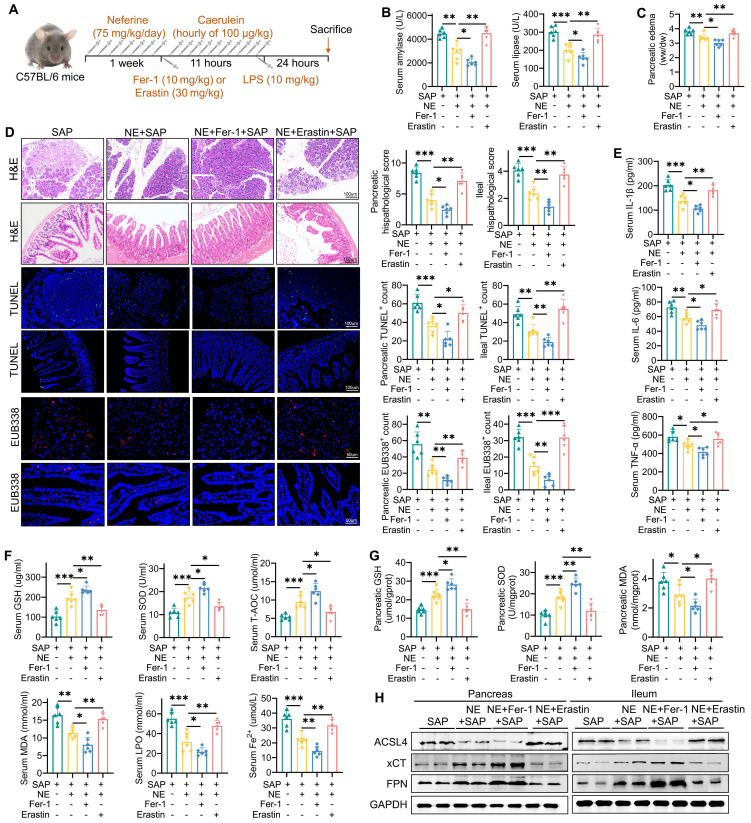
Upregulation of ferroptosis reversed the protective effects of neferine on pancreatic and ileal injury. (A) Experimental schedule for SAP establishment. (B) Serum levels of amylase and lipase. (C) Pancreatic edema assessed by the wet-to-dry ratio. (D) H&E staining and corresponding histologic scores; Immunofluorescence staining of TUNEL and quantification of TUNEL^+^ cells; Positive hybridization signals of total bacteria detected by the EUB338 probe, with quantification of EUB338-positive bacteria in the per field. (E) Serum levels of IL-1β, IL-6, and TNF-α. (F) Serum levels of GSH, SOD, T-AOC, MDA, LPO, and Fe^2+^. (G) Pancreatic levels of SOD, GSH, and MDA. (H) Western blot of ACSL4, xCT, and FPN protein levels in the pancreas and ileum. NE, Neferine; Data were expressed as mean ± SD; n = 6 in each group. *P < 0.05, **P < 0.01, ***P < 0.001.

We further explored the role of ferroptosis in the protective effects of neferine on pancreatic and intestinal injury by utilizing the ferroptosis inhibitor Fer-1 and the ferroptosis agonist erastin (Fig. 4A). Compared to SAP mice treated with neferine alone, the combination of neferine and erastin significantly elevated amylase and lipase levels (Fig. 4B), exacerbated pancreatic edema (Fig. 4C), and worsened pancreatic and ileal injury scores (Fig. 4D). Erastin treatment also significantly reversed the reductions in pro-inflammatory cytokines (Fig. 4E, S7A-D), apoptosis (Fig. 4D), neutrophil and M1 macrophage infiltration (Fig. S8A, S8B) in SAP mice receiving neferine treatment. Additionally, neferine plus erastin treatment reduced the expression of intestinal MUC2 and lysozyme (Fig. S9A), compromised intestinal barrier function (Fig. S9B), and promoted the translocation of intestinal flora into both the ileum and pancreas in SAP mice (Fig. 4D).

Erastin significantly negated protective effects of neferine on ferroptosis indicators, such as GSH, SOD, T-AOC, MDA, LPO, and Fe²⁺ (Fig. 4F, 4G, S9C). Furthermore, erastin significantly suppressed xCT and FPN expression, increased ACSL4 expression (Fig. 4H, S9D, S9E) in pancreatic and ileal tissues. Consequently, erastin nearly abolished the therapeutic benefits of neferine in SAP mice. Conversely, the combination of neferine and Fer-1 demonstrated the highest therapeutic efficacy (Fig. 4, S7-9).

### Neferine protects against SAP-induced ferroptosis in an Nrf2-dependent manner

**Figure 5:**
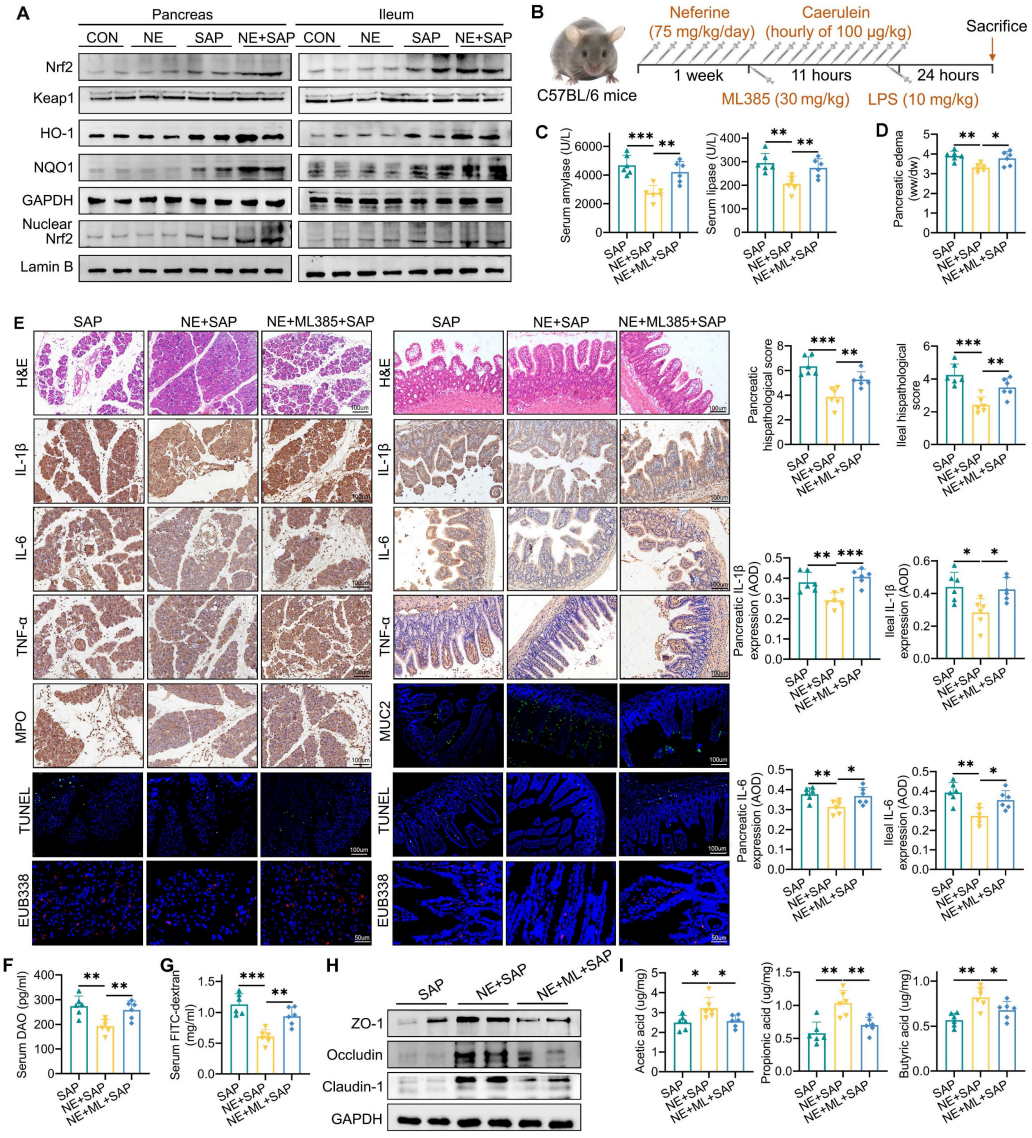
Neferine protects against SAP-induced ferroptosis in an Nrf2-dependent manner. (A) Western blot analysis of total Nrf2, Keap1, HO-1, NQO-1, and nuclear Nrf2 protein levels in the pancreas and ileum. (B) Experimental schedule for SAP establishment. (C) Serum levels of amylase and lipase. (D) Pancreatic edema assessed by the wet-to-dry ratio. (E) H&E staining and corresponding histologic scores; Immunohistochemical staining of IL-1β, IL-6, and TNF-α, along with average optical density calculations; Immunohistochemical staining of pancreatic MPO; Immunofluorescence staining of MUC2, and TUNEL; Positive hybridization signals of total bacteria detected by the EUB338 probe, with quantification of EUB338-positive bacteria in the per field. (F) Serum DAO levels measured by ELISA. (G) Serum FITC-dextran levels measured by ELISA. (H) Western blot of ZO-1, occludin, and claudin-1 protein levels in the ileum. (I) Fecal levels of acetic acid, propionic acid, and butyric acid. AOD, average optical density; NE, Neferine; ML, ML385. Data were expressed as mean ± SD; n = 6 in each group. *P < 0.05, **P < 0.01, ***P < 0.001.

Previous studies have shown that Nrf2 plays a crucial role in suppressing ferroptosis by regulating key molecules in the ferroptosis cascade, including FPN, xCT, and GPX4 32, 33. Based on this, we hypothesize that neferine may regulate ferroptosis through the Nrf2 pathway. To investigate this, we analyzed protein expression levels and found that neferine treatment significantly increased the expression of total Nrf2, nuclear Nrf2, HO-1, and NQO1 in SAP mice (Fig. 5A, S10A, S10B).

To further investigate the precise role of Nrf2 signaling in the protective effects of neferine against ferroptosis, the Nrf2 inhibitor ML385 was used (Fig. 5B). Our findings showed that ML385 effectively negated the protective effects of neferine against pancreatic and ileal injury, as evidenced by elevated amylase and lipase levels (Fig. 5C), increased edema (Fig. 5D), and worsened histopathological scores (Fig. 5E). Additionally, significantly higher levels of IL-1β, IL-6, TNF-α (Fig. 5E, Fig. S11A-D), apoptosis (Fig. 5E, S12A), neutrophil and M1 macrophage infiltration (S12B-D), and intestinal flora translocation (Fig. 5E-G, S13A), along with decreased levels of IL-10 (Fig. S11A), M2 macrophage infiltration (Fig. S12D), MUC2 (Fig. 5E, S12E), lysozyme (Fig. S12F), intestinal barrier proteins (Fig. 5H, S13B), and SCFAs (Fig. 5I, S13C) were observed in the serum, pancreas, and ileum of SAP mice receiving both neferine and ML385 treatments, compared to those treated with neferine alone.

**Figure 6:**
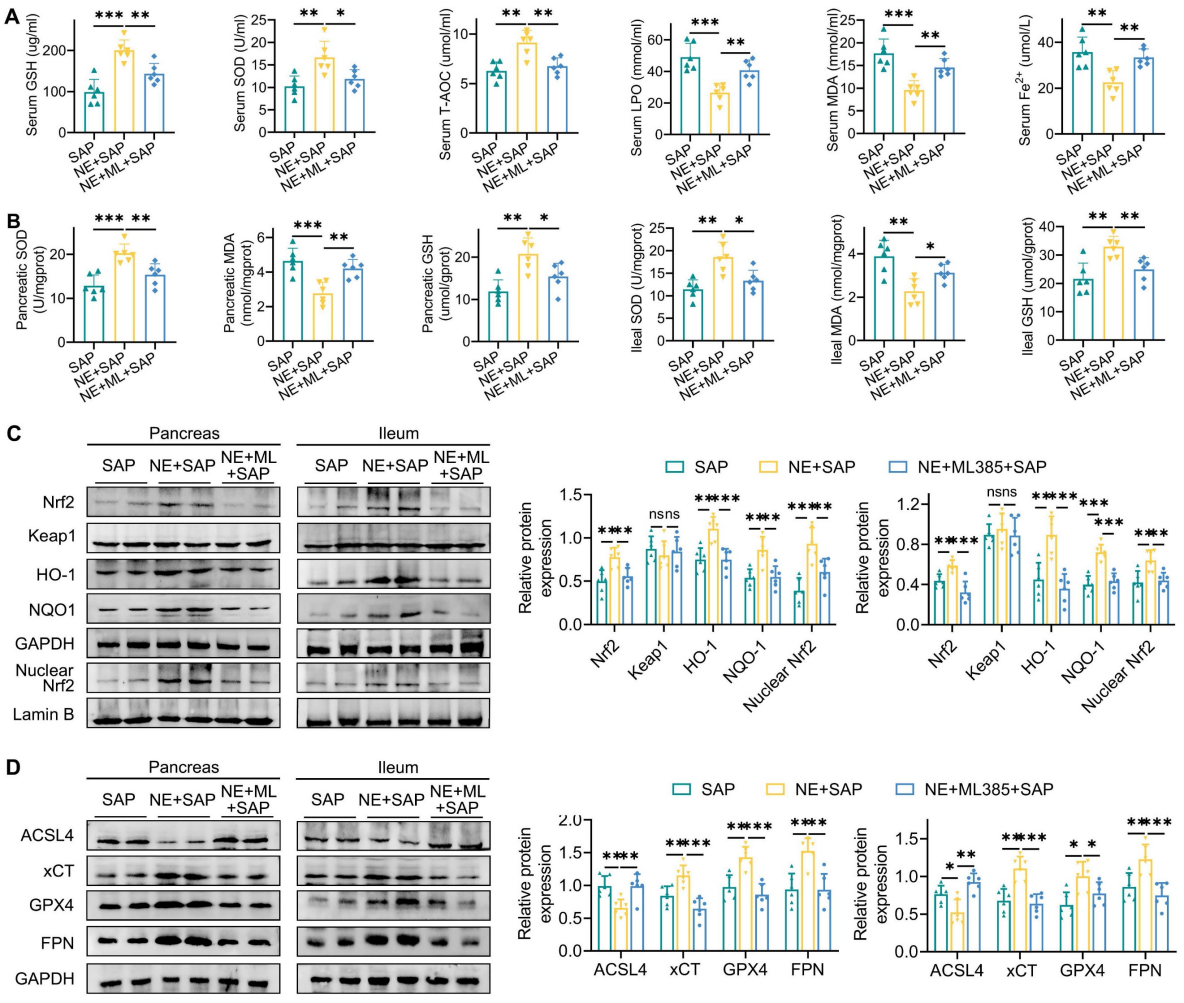
Inhibition of Nrf2 reverses the inhibitory effects of neferine on ferroptosis. (A) Serum levels of GSH, SOD, T-AOC, MDA, LPO, and Fe^2+^. (B) Pancreatic and ileal levels of MDA, SOD, and GSH. (C) Western blot analysis and corresponding relative gray values of Nrf2, Keap1, HO-1, NQO-1, and nuclear Nrf2 protein levels in the pancreas and ileum. (D) Western blot analysis and relative gray values of ACSL4, xCT, GPX4, and FPN protein levels in the pancreas and ileum. NE, Neferine; ML, ML385. Data are presented as mean ± SD, with n = 6 per group. *P < 0.05, **P < 0.01, ***P < 0.001.

Additionally, ML385 effectively reversed the beneficial effects of neferine on ferroptosis markers, including GSH, SOD, T-AOC, MDA, LPO, and Fe²⁺ (Fig. 6A, 6B). Furthermore, while neferine increased the expression of pancreatic and intestinal total Nrf2, nuclear Nrf2, HO-1, NQO1, xCT, GPX4, and FPN, and decreased ACSL4 expression, these effects were abolished following ML385 treatment (Fig. 6C, 6D, S13D).

In summary, neferine alleviates SAP and SAP-associated intestinal injury by synergistically inhibiting ferroptosis through iron efflux mediated by the NRF2/FPN axis and ferroptosis defense mediated by the NRF2/xCT/GPX4 axis.

### Neferine inhibits ferroptosis by upregulating the Nrf2 pathway *in vitro*

**Figure 7:**
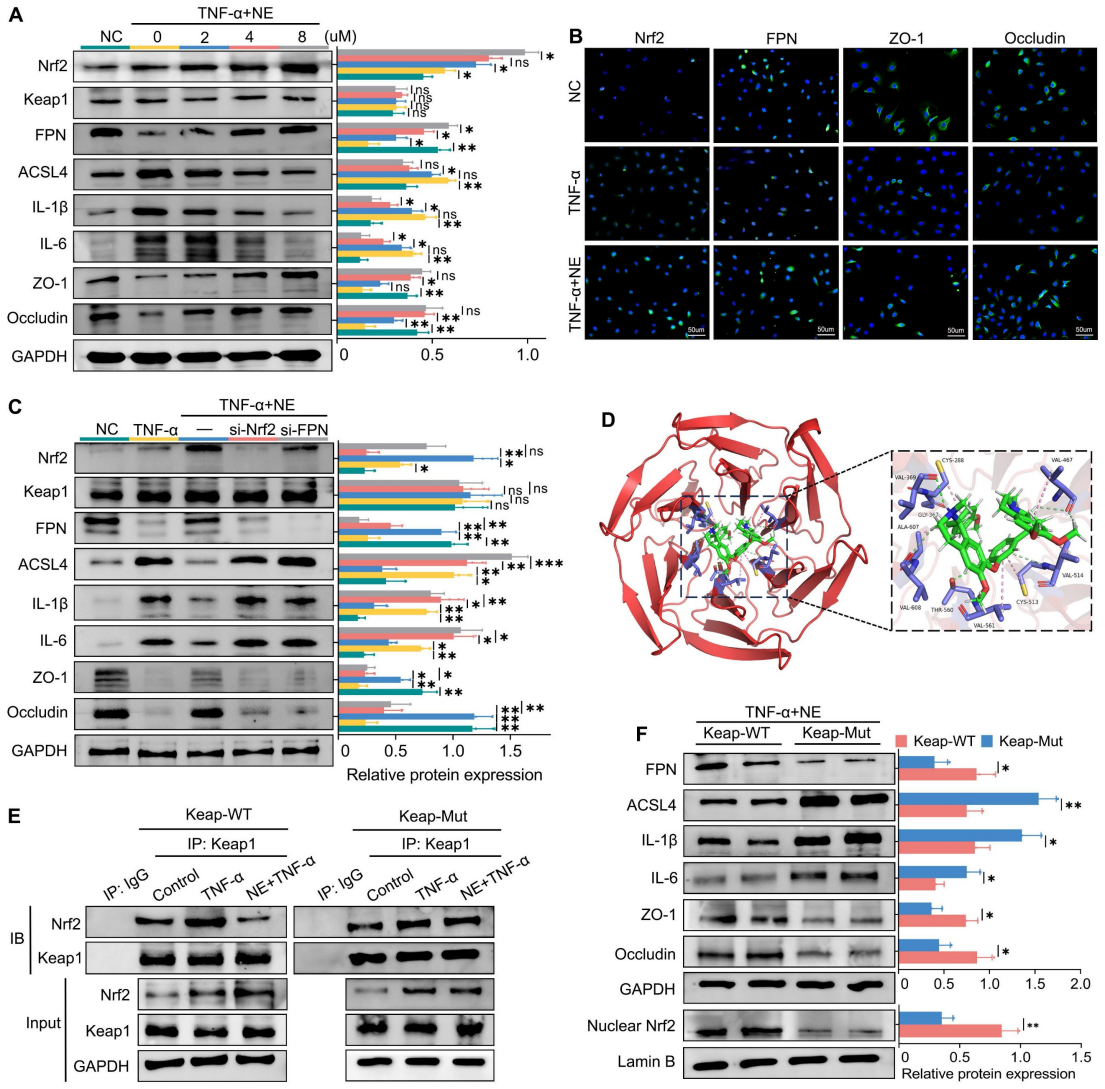
Neferine inhibits ferroptosis by upregulating the Nrf2 pathway *in vitro*. (A) Western blot analysis was performed to detect the expression of Nrf2, Keap1, FPN, ACSL4, IL-1β, IL-6, ZO-1, and Occludin in IEC-6 cells subjected to different neferine treatments. (B) Immunofluorescence staining was conducted to visualize Nrf2, FPN, ZO-1, and Occludin in IEC-6 cells; The neferine concentration used is 4 μM. (C) Western blot analysis was performed to measure the expression of Nrf2, Keap1, FPN, ACSL4, IL-1β, IL-6, ZO-1, and Occludin in IEC-6 cells treated with si-Nrf2 and si-FPN. (D) Amino acid binding sites of neferine within the crystal structure of the Keap1 complex. (E) Co-immunoprecipitation analysis illustrating the interaction between Keap1 and Nrf2 in wild-type and Keap1-mutant IEC-6 cells. (F) Western blot analysis was performed to measure the expression of nuclear Nrf2, FPN, ACSL4, IL-1β, IL-6, ZO-1, and Occludin in wild-type and Keap1-mutant IEC-6 cells. NE, Neferine; Data were expressed as mean ± SD; n = 6 in each group. *P < 0.05, **P < 0.01, ***P < 0.001.

Systemic inflammation triggers a cascade of events that leads to elevated cytokine levels, particularly TNF-α, which plays a crucial role in the pathogenesis of SAP-related intestinal injury. Thus, TNF-α was selected as the target for *in vitro* experiments. Initially, we assessed the impact of neferine on cell viability using the CCK8 assay. IEC-6 cells were treated with various concentrations of neferine (1, 2, 4, 8, 16, 32, or 64 μM). As shown in Fig. S14, 16 μM neferine led to a reduction in cell viability, whereas concentrations between 1 and 8 μM did not induce cytotoxic effects on IEC cells. Western blot analysis revealed that TNF-α stimulation resulted in significant increases in ACSL4, IL-1β, and IL-6 levels while reducing the levels of FPN, ZO-1, and occludin (Fig. 7A). However, neferine treatment upregulated the levels of Nrf2, FPN, ZO-1, and occludin and downregulated the levels of ACSL4, IL-1β, and IL-6 with the most prominent effects observed at a concentration of 8 μM (Fig. 7A).

IF analysis of IEC-6 cells demonstrated that neferine enhanced the expression of FPN, ZO-1, and occludin and promoted the translocation of Nrf2 from the cytoplasm to the nucleus (Fig. 7B). Moreover, knocking down either Nrf2 or FPN in IEC-6 and AR42J cells reversed the protective influence of neferine (Fig. 7C, S15A). In conclusion, *in vitro* experiments confirm that neferine exerts its protective effects through the Keap1/Nrf2-ferroptosis axis.

Additionally, we further explored the relationship between pancreatitis and intestinal injury using IEC-6 and AR42J cells. We cultured IEC-6 cells with the supernatant from TNF-α-stimulated AR42J cells, using the supernatant from untreated AR42J cells as a control. Our results showed that inflammatory factors secreted by damaged AR42J cells promoted IEC-6 cell injury and upregulated ferroptosis levels (Fig. S15B).

### Neferine promotes Nrf2 nuclear translocation by competitively binding to the Cys-288 site of Keap1

Keap1 suppresses Nrf2 activity under basal conditions; however, upon exposure to various stressors, Nrf2 is released from Keap1-mediated repression 34. Given that Keap1 did not exhibit significant changes in Nrf2 activation, we investigated whether neferine interacts with Keap1 to modulate Nrf2 activity. Molecular docking analysis showed that the binding energy between neferine and Keap1 was -8.621 kcal/mol (Fig. 7D), which is below the -5.0 kcal/mol threshold, indicating a potential interaction 35.

In the crystal structure of the Keap1 complex, neferine forms hydrogen bonds and hydrophobic interactions with its surrounding amino acids, including Ala-607, Cys-288, Cys-513, Gly-367, Thr-560, Val-608, Val-561, Val-514, Val-467, Val-369 (Fig. 7D). Interestingly, Cys-288 has also been identified as a binding site for Keap1 and Nrf2 36.

To determine whether neferine activates Nrf2 through competitive binding at the Cys-288 site of Keap1, we transfected IEC-6 cells with either wild-type or Cys-288 mutant Keap1 plasmids. Co-immunoprecipitation assays demonstrated that the ability of neferine to enhance Keap1-Nrf2 dissociation was significantly reduced in Keap1-mutant IEC-6 cells (Fig. 7E). Additionally, the expressions of IL-1β, IL-6, and ACSL4 in Keap1-mutant IEC-6 and AR42J cells were significantly higher compared to wild-type IEC-6 cells, while the expressions of nuclear Nrf2, FPN, ZO-1, and occludin were significantly decreased (Fig. 7F, S15C). These findings confirm that neferine promotes Keap1-Nrf2 dissociation and enhances Nrf2 nuclear translocation by competitively interacting with the Cys-288 site of Keap1.

## Discussion

**Figure 8:**
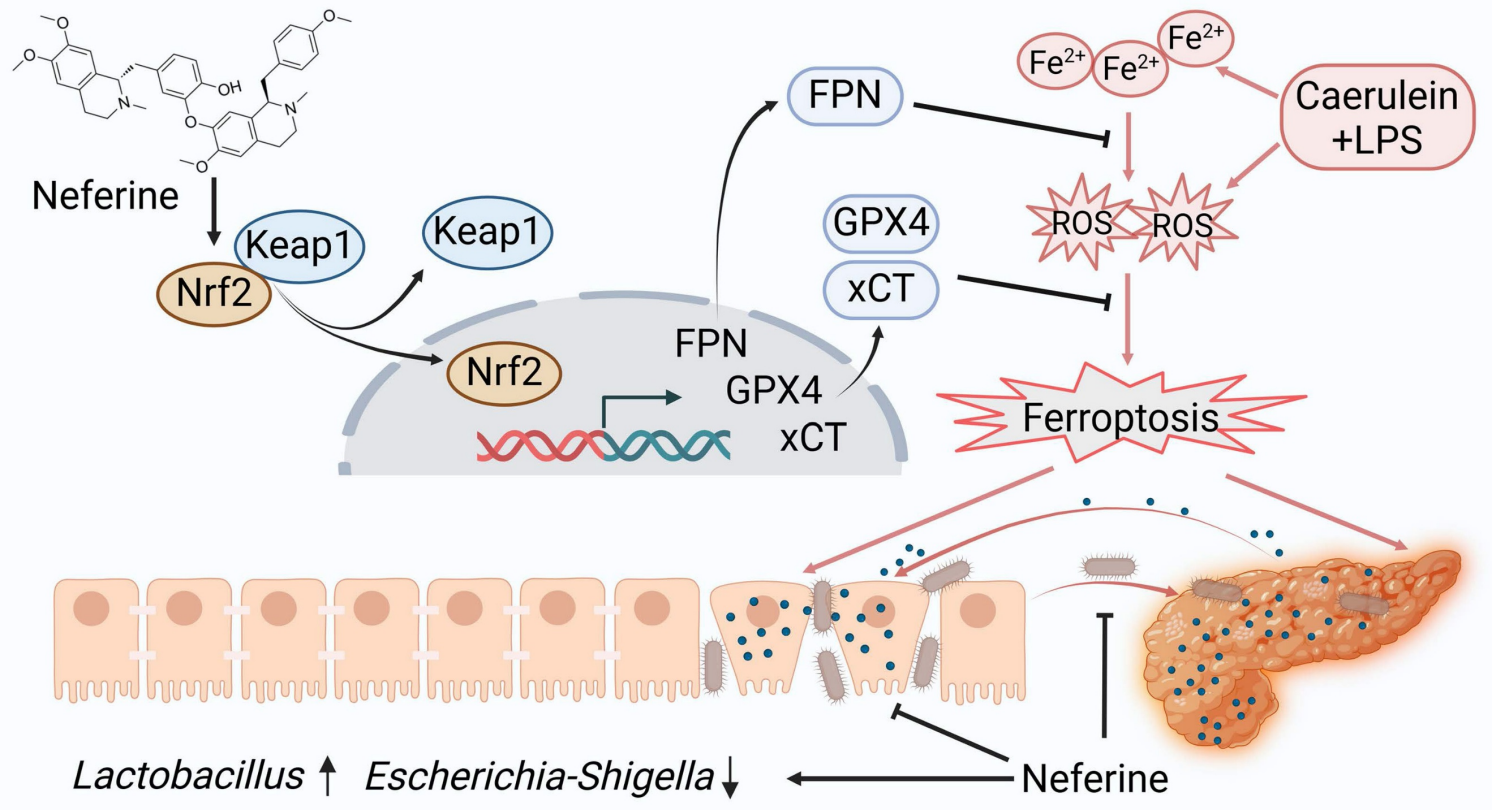
A proposed model illustrates the mechanism by which neferine protects against SAP and its associated intestinal injury. In SAP, iron metabolism becomes dysregulated, and ROS production increases. Neferine administration not only upregulates Nrf2 but also promotes the dissociation of Keap1-Nrf2 and enhances Nrf2 nuclear translocation by competitively binding to the Cys-288 site of Keap1. This activation strengthens the Nrf2/FPN and Nrf2/xCT/GPX4 axes, thereby preventing ferroptosis and ultimately protecting against pancreatic and intestinal injury in SAP mice. Additionally, neferine helps restore intestinal homeostasis by increasing the abundance of beneficial *Lactobacillus*, reducing harmful *Escherichia-Shigella*, and inhibiting intestinal flora migration. Ultimately, neferine administration disrupts the positive feedback loop in pancreatitis, where pancreatic inflammation triggers systemic inflammation, leading to intestinal barrier damage, subsequent gut microbiota translocation, and further exacerbation of pancreatic injury.

This study examined the protective effects of neferine in experimental SAP from multiple perspectives. Our findings indicate that neferine supplementation not only alleviates pancreatic and intestinal damage by inhibiting ferroptosis (Fig. 8) but also preserves intestinal barrier integrityand prevents bacterial translocation through modulation of the gut microbiota and enhancement of SCFAs production. Thus, the anti-inflammatory and antioxidant properties of neferine, combined with its ability to maintain gut homeostasis, collectively contribute to the mitigation of SAP. This is the first report demonstrating the efficacy of neferine in experimental SAP.

During SAP, damage to pancreatic acinar cells, along with the infiltration of activated inflammatory cells, results in the release of excessive ROS and a reduction in antioxidant levels. This imbalance promotes ferroptosis, which further exacerbates the initial injury 37. Previous studies have shown that ROS-induced damage in pancreatitis can be mitigated through activation of the Keap1/Nrf2 signaling pathway 38. Recent work by Wu *et al.* suggests that neferine ameliorates ischemic brain injury by enhancing Nrf2 nuclear translocation 39. Moreover, prior work has shown that monomeric compounds and proteins can activate the Nrf2 pathway through competitive binding with Keap1 40, 41. Our investigation reveals that neferine upregulates Nrf2 expression and promotes its nuclear translocation by competitively binding to the Cys-288 site on Keap1.

In the present study, transcriptomic analysis revealed that neferine significantly reduces ferroptosis in SAP mice. Circulating Fe³⁺ binds to transferrin for transport, and upon entering the cell via transferrin receptor 1, it is reduced and released into the cytoplasmic labile iron pool (LIP) 6. Excess iron is stored in ferritin. The intracellular LIP, primarily in the form of Fe²⁺, is unstable and highly reactive 6. Fe²⁺ can generate hydroxyl radicals through the Fenton reaction, which react with polyunsaturated fatty acids in the cell and plasma membranes, producing lipid ROS and leading to ferroptosis. In our study, we found that Fe²⁺ levels were significantly increased in the pancreas, intestine, and serum of SAP mice. In our study, we found that SAP mice exhibited significantly elevated levels of Fe²⁺ in the pancreas, intestine, and serum. Neferine regulates iron metabolism through the Nrf2/FPN axis and exerts antioxidant effects via the Nrf2/xCT/GPX4 axis, thereby inhibiting ferroptosis and providing protective effects.

Disruption of the intestinal barrier in SAP facilitates the movement of bacteria and endotoxins, playing a critical role in secondary tissue damage and contributing to the onset of SIRS 42. An increasing body of research has demonstrated that the preservation of the intestinal barrier is strongly associated with the clinical outcome of AP 43. In this study, the neferine-treated mice showed a decrease in apoptotic IECs and an increase in the expression of tight junction proteins (ZO-1, occludin, and claudin-1), underscoring the protective role of neferine in maintaining intestinal barrier integrity, reducing intestinal permeability, and preventing bacterial translocation.

Consistent with earlier findings that reduced microbiome diversity is commonly observed in SAP patients, our study identified significant changes in both the abundance and composition of the gut microbiota associated with SAP 44, 45. Importantly, neferine treatment significantly maintained the diversity of microbiota in SAP mice and increased the abundance of beneficial bacteria (*Lactobacillus*, *Lachnospiraceae_NK4A136_group*, *Alistipes*, *Prevotellaceae_UCG-001*, and *Eubacterium_xylanophilum_group*) and decreased the abundance of pathogenic bacteria (*Escherichia-Shigella*). *Lactobacillus*, a widely recognized probiotic, plays a crucial role in preventing pathogen colonization in the intestine, inhibiting infections, supporting microecological balance, enhancing immune function, synthesizing essential amino acids and vitamins, and suppressing endotoxin production 46. Recent research by Zhou *et al.* demonstrated that norharman, a tryptophan metabolite produced by cultured *Lactobacillus*, alleviates AP by acting as an antagonist of histone deacetylases 47.

*Lactobacillus*,* Lachnospiraceae_nk4a136_group*,* and Eubacterium xylanophilum_group* produce SCFAs, which support gut health by providing energy to epithelial cells, maintaining barrier integrity, and reducing inflammation 48-51. SCFAs also demonstrate anti-inflammatory properties in pancreatitis 52. In this study, the higher concentration of SCFAs in the neferine-treated group suggests that the protective effect of neferine may be associated with its role in SCFA synthesis. *Prevotellaceae_UCG-001* plays a role in inhibiting pathogenic bacterial growth and maintaining intestinal stability 53. Alistipes, a commensal bacterium belonging to the genus Bacteroides, has been shown to suppress pro-inflammatory cytokine production and alleviate disease activity in an ulcerative colitis mouse model when its levels are increased 54. In contrast, *Escherichia-Shigella*, a gram-negative pathogenic bacterium, contributes to intestinal inflammation by infiltrating epithelial cells and promoting the release of IL-1β 55. Studies have also indicated that SAP leads to significant changes in gut microbiota composition, including a marked increase in *Escherichia-Shigella* abundance, as demonstrated through 16S rRNA sequencing 55.

## Conclusion

This study is the first to demonstrate the protective role of neferine in SAP and elucidate its underlying mechanisms. Our findings indicate that neferine mitigates SAP by regulating Nrf2/FPN and Nrf2/xCT/GPX4 axes. Additionally, its protective effects are linked to modulation of the gut microbiota and enhanced SCFAs synthesis. Overall, our results provide valuable insights into the therapeutic potential of neferine in preventing SAP and offer compelling evidence for its consideration as a promising therapeutic approach for SAP management.

## Supplementary Material

Supplementary materials and methods, figures.
