# Supplementary material for: Neferine Ameliorates Severe Acute Pancreatitis-Associated Intestinal Injury by Promoting NRF2-mediated Ferroptosis
Source: Int J Biol Sci. 2025 Apr 28;21(7):3247–61. doi: 10.7150/ijbs.112888 (PMC12080392; doi:10.7150/ijbs.112888)
Supplement: Supplementary file 1 — Supplementary materials and methods, figures. [file ijbsv21p3247s1.pdf]

Neferine Ameliorates Severe Acute Pancreatitis-Associated Intestinal Injury by Promoting FPN-Mediated Iron Export and Inhibiting Ferroptosis

Supplementary methods

1. Animals

7-week-old male C57BL/6 mice, weighing 20–22 g, were purchased from Shulaibao Biotechnology Co., Ltd. (Wuhan, China) and housed at the Animal Facility of Renmin Hospital of Wuhan University. The mice were maintained under specific pathogen-free conditions, with a controlled temperature of 23–25 °C and a 12-hour light/dark cycle. Prior to the experiments, the mice underwent a one-week acclimatization period to adjust to the laboratory environment.

2. Mouse Serum Sample Acquisition

The blood samples were centrifuged at 3000 × g for 15 minutes after coagulation at room temperature for 25 minutes. Following centrifugation, the serum was collected and stored at -80°C until further analysis.

3. Serum amylase, lipase activity, and serum biochemical indices

Using the serum lipase assay kit (Jiancheng Bioengineering Institute, Nanjing, China) and serum amylase assay kit (Changchun Huili Biotech Co., Ltd, Changchun, China), the activities of both enzymes were measured, following the guidelines given in their

respective manuals. Additionally, biochemical markers such as alanine aminotransferase (ALT), aspartate aminotransferase (AST), creatinine, and Urea were analyzed using an automated biochemical analyzer.

4. Determination of pancreatic edema

Pancreatic edema was assessed by calculating the ratio of wet to dry weight. Wet weight was measured using freshly excised pancreatic tissue, which was then vacuum-dried. The dry weight was determined after the samples were dehydrated at 80°C for 48 hours.

5. Histological examination

The tissues were fixed in 4% paraformaldehyde, followed by dehydration with ethanol and embedding in paraffin. Once embedded, the tissues were sectioned into 5 μm slices using a Skiving Machine Slicer and stained with hematoxylin and eosin (HE). Morphological alterations were examined using light microscopy (Olympus) at × 200 magnification. Pathological changes in the ileum were assessed according to Chiu's criteria (Chiu et al., 1970), while pancreatic histological modifications were evaluated based on the Schmidt criteria (Shimizu et al., 2000).

6. Fluorescence in situ hybridization (FISH)

Bacterial translocation was evaluated using FISH, following previously described protocols (Zheng et al., 2019). In brief, tissue samples from the distal ileum and pancreas were subjected to dewaxing, which involved 70 minutes at 65 °C, two 10-minute

treatments with 100% xylene, and a 5-minute wash with 100% ethanol. After air drying, the specimens were incubated with specific probes (EUB338: 5’-Cy3-GCTGCCTCCCGTAGGAGT-3’) in a humidified chamber at 52 °C for 18 hours. The sections were then washed, counterstained with DAPI, and analyzed under an Olympus fluorescence microscope.

7. Immunohistochemical (IHC), immunofluorescence (IF), and TUNEL staining

The paraffin-embedded sections underwent a series of treatments, including baking, dewaxing, hydration, and antigen retrieval using Tris-EDTA buffer. Following these steps, the slides were incubated with a 3% hydrogen peroxide solution for 15 minutes to block endogenous peroxidase activity. Eukaryotic cell membrane permeability was enhanced by applying Triton X-100 (Servicebio, Wuhan, China). Primary antibodies targeting IL-1β (1:100, Baijia, Rabbit, IMB0001), IL-6 (1:100, Proteintech, Mouse, 66146-1-Ig), TNF-α (1:100, Proteintech, Mouse, 60291-1-Ig), MPO (1:100, Proteintech, Rabbit, 22225-1-AP), and ACSL4 (1:100, Proteintech, Rabbit, 22401-1-AP) were applied and allowed to incubate overnight at 4°C. A secondary antibody was subsequently applied. The resulting staining was visualized using 3,5-diaminobenzidine (DAB). All sections were analyzed under an Olympus light microscope in a blinded manner, and images were captured accordingly. The integrated optical density/area (AOD) of the immunostained sections was quantitatively assessed using Image-Pro Plus 6.0 software (Media Cybernetics Inc, Bethesda, USA).

For immunofluorescence staining, an additional blocking step was performed with 10%

donkey serum for 1 hour, followed by overnight incubation of the slides with primary antibodies at 4°C. The following primary antibodies were used for single staining: Ly6G (1:100, Santa, Mouse, SC53515), lysozyme (1:100, abclonal, Rabbit, A0641), MUC2 (1:100, abclonal, Rabbit, A14659), ZO-1 (1:100, Proteintech, Rabbit, 21773-1-AP), Occludin (1:100, Proteintech, Rabbit, 27260-1-AP), GPX4 (1:100, Abclonal, Rabbit, A13309), Nrf2 (1:100, Abclonal, Rabbit, A11159), F4/80 (1:100, Proteintech, Rabbit, 28463-1-AP), iNOS (1:100, Abcam, Mouse, AB3523), and CD206 (1:100, Abcam, Mouse, AB64693). After washing, the slides were incubated for one hour with fluorescein-labeled secondary antibodies and counterstained with DAPI for five minutes to visualize the nuclei.

Apoptosis in pancreatic and intestinal epithelial cells was evaluated using the One Step TUNEL Apoptosis Detection Kit (Green Fluorescent) (Beyotime, China) according to the manufacturer's guidelines. All slides were examined using an Olympus fluorescence microscope, and the cell positivity rate was subsequently quantified.

8. Enzyme-linked immunosorbent assay (ELISA)

Serum levels of IL-1β (MultiSciences (Lianke) Biotech Co., Ltd., Huangzhou, China), TNF-α (MultiSciences (Lianke) Biotech Co., Ltd.), IL-6 (Elabscience Biotechnology Co., Ltd., Wuhan, China), IL-10 (Elabscience Biotechnology Co., Ltd.), malondialdehyde (MDA) (Beijing Solarbio Science & Technology Co., Ltd.), glutathione (GSH) (Solarbio Science & Technology Co., Ltd.), superoxide dismutase (SOD) (Solarbio Science & Technology Co., Ltd.), lipid peroxidation (LPO) (Solarbio

Science & Technology Co., Ltd.), total antioxidant capacity (T-AOC) (Solarbio Science & Technology Co., Ltd.), and Fe^{2+} (Solarbio Science & Technology Co., Ltd.) were measured using appropriate assay kits, following the manufacturers' guidelines. Pancreas and intestinal tissue levels of malondialdehyde (MDA) (Jiancheng Bioengineering Institute), glutathione (GSH) (Jiancheng Bioengineering Institute), and superoxide dismutase (SOD) (Jiancheng Bioengineering Institute) were measured using appropriate assay kits, following the manufacturers' guidelines. The activities of both enzymes were assessed using the serum lipase assay kit (Jiancheng Bioengineering Institute) and the serum amylase assay kit (Changchun Huili Biotech Co., Ltd), in accordance with the protocols provided in their respective manuals.

9. Short-chain fatty acid (SCFA) analysis

The concentrations of SCFA in fecal samples were determined using GC-MS (Shimadzu, Kyoto, Japan), following established methods (Shang et al., 2016). In brief, 500 μL of a saturated NaCl solution was mixed with 50 mg of fecal material to achieve homogenization. The resulting mixture was then acidified with 40 μL of 10% sulfuric acid. To facilitate SCFA extraction, 800 μL of diethyl ether was subsequently added. The samples were centrifuged at 14,000 rpm for 15 minutes at 4°C, and the supernatants were analyzed by GC-MS.

10. Co-immunoprecipitation and immunoblot analysis

Pancreatic and ileal tissues, along with IEC-6 cells, were lysed in RIPA buffer

supplemented with a complete protease inhibitor cocktail (Servicebio, Wuhan, China). The resulting mixture was subjected to centrifugation at 4 °C and 12,000 × g for 20 minutes to isolate the proteins. Following a 10-minute heat treatment at 100 °C, the protein extracts were resolved by SDS-PAGE and subsequently transferred to a nitrocellulose membrane. After blocking with 5% fat-free milk for 2 hours at room temperature, the membrane was incubated overnight at 4 °C with primary antibodies. This was followed by incubation with HRP-conjugated secondary antibodies. The primary antibodies used were: IL-1β (1:1000, Baijia, Rabbit, IMB0001), IL-6 (1:1000, Proteintech, Mouse, 66146-1-Ig), TNF-α (1:1000, Proteintech, Mouse, 60291-1-Ig), ZO-1 (1:1000, Proteintech, Rabbit, 21773-1-AP), Occludin (1:1000, Proteintech, Rabbit, 27260-1-AP), Claduin-1 (1:1000, Proteintech, Rabbit, 28674-1-AP), ACSL4 (1:1000, Proteintech, Rabbit, 22401-1-AP), xCT (1:1000, Abmart, Rabbit, T57046), GPX4 (1:1000, Abclonal, Rabbit, A13309), FPN (1:1000, Proteintech, Rabbit, 26601-1-AP), Nrf2 (1:1000, Abclonal, Rabbit, A11159), Keap1 (1:1000, Baijia, Rabbit, IPB0177), HO-1 (1:1000, Baijia, Mouse, IMB0839), and NQO-1 (1:1000, Abmart, Rabbit, T56710F). Protein bands were visualized using an enhanced chemiluminescence detection kit (Wuhampmek Biotechnology Co., Ltd., Wuhan, China). Finally, the intensity of each band was measured using ImageJ software and normalized to the GAPDH (1:1000, Proteintech, Mouse, 60004-1-Ig) or Lamin B1 (1:1000, Proteintech, Rabbit, 12987-1-AP) band density.

For Co-immunoprecipitation, proteins were immunoprecipitated with anti-Keap1 antibody (Baijia, IPB0177). The precleared protein A/G magnetic beads (HY-K0202,

MedChemExpress, Shanghai, China) were incubated with immunocomplexes and washed with the lysis buffer.

11. Quantitative real-time PCR (qRT-PCR)

RNA was isolated from tissue samples using the RNA extraction kit (Servicebio, Wuhan, China), and its concentration was quantified with a Thermo NanoDrop2000 spectrophotometer at 260 nm. cDNA synthesis was carried out using the SweScript One-Step RT-PCR Kit (Servicebio, Wuhan, China). Quantitative PCR was conducted with the Bio-Rad SYBR Green Supermix and gene-specific primers on the Bio-Rad CFX Connect Real-Time System (CA, USA). β-actin was used as the internal control.

(IL-1β: F, GCAACTGTTCCTGAACTCAACT; R, ATCTTTTGGGGTCCGTCAACT
IL-6: F, TAGTCCTTCCTACCCCAATTTCC; R, TTGGTCCTTAGCCACTCCTTC
TNF-α: F, CCCTCACACTCAGATCATCTTCT; R, GCTACGACGTGGGCTACAG
β-actin: F, CCCAGGCATTGCTGACAGG; R, TGGAAGGTGGACAGTGAGGC
TFR1: F, CTTCGCAGGCCAGTGCT; R, CTTGCCGAGCAAGGCTAAAC
FTH1: F, CTGGAACTGCACAAACTGGC; R, CTCTCATCACCGTGTCCCAG
FTL: F, CTCCTTGCCCGGGACTTAGA; R, ACTTGTAAAGGCGGCTGGAA
FPN: F, TCCAACCCGCTCCCATAAG; R, AAAGCTGTCACGGGGTCTTC
NCOA4: F, AGATACATCTGCTCTGCGCC; R, TACAGCTGTGCCACTGGATG
xCT: F,GTCATCGGATCAGGCATCTT; R, CATAGGACAGGGCTCCAAAA
GPX4: F,CCCGATACGCTGAGTGTGGTTTG; R, TCTTCGTTACTCCCTGGCTCC
TG)

12. 16S rRNA gene sequences

DNA was extracted from mouse fecal samples using the CTAB method (Fujimura et al., 2016). To determine the purity and concentration of the DNA, agarose gel electrophoresis (1% agarose gels) was performed. The hypervariable 16SV34 region of the 16S gene was amplified using barcode-labeled specific primers. PCR reactions were conducted with 15 μL of Phusion® High-Fidelity PCR Master Mix (New England Biolabs), 2 μM of each forward and reverse primer (341 (5'-CCTAYGGGRBGCASCAG-3') and 806 (3'-GGACTACNNGGGTATCTAAT-5')), and approximately 10 ng of template DNA. The thermal cycling protocol was as follows: initial denaturation at 98°C for 1 minute; 30 cycles of denaturation at 98°C for 10 seconds, annealing at 50°C for 30 seconds, and elongation at 72°C for 30 seconds; followed by a final elongation step at 72°C for 5 minutes. Fecal DNA extraction and PCR amplification were conducted in a UV-sterilized biological safety cabinet, with a minimum exposure time of 60 minutes to prevent contamination with environmental DNA.

The PCR products were combined with an equal volume of 1x loading buffer containing SYBR Green dye and subjected to electrophoresis on 2% agarose gels for visualization. PCR product purification was performed using the Qiagen Gel Extraction Kit (Qiagen, Germany) according to the manufacturer's instructions. Next, sequencing libraries were constructed using the TruSeq® DNA PCR-Free Sample Preparation Kit (Illumina, USA), incorporating index codes. Library quality was evaluated with the Qubit® 2.0 Fluorometer (Thermo Scientific). Finally, sequencing was conducted on an Illumina

NovaSeq 6000 PE250 platform, generating 250 bp paired-end reads.

The sequencing data from each sample were segregated based on Barcode and PCR amplified primer sequences. The reads corresponding to each sample were then merged using FLASH (V1.2.7, http://ccb.jhu.edu/software/FLASH/) (Magoč and Salzberg, 2011) after the removal of the Barcode and primer sequences. This generated the raw Tags data (Raw Tags). Following this, the Raw Tags underwent rigorous filtering with fastp software (Bokulich et al., 2013) to produce high-quality Tags data (Clean Tags). The resulting Tags were then compared to the species annotation database (https://github.com/torognes/vsearch/) (Rognes et al., 2016) to identify and eliminate chimera sequences (Haas et al., 2011). The final dataset, comprising the Effective Tags, was obtained after this step.

The DADA2 method is primarily designed for noise reduction (Callahan et al., 2016), emphasizing dereplication or 100% similarity clustering, rather than traditional similarity-based clustering. Sequences that are de-duplicated by DADA2 are referred to as Amplicon Sequence Variants (ASVs) or feature sequences, which are analogous to OTU sequences. The resulting table, which outlines the abundance of these sequences across samples, is known as the feature table (comparable to the OTU table). In comparison to the traditional OTU-based approach, DADA2 offers superior sensitivity and specificity, allowing for the identification of true biological variants that are often missed by OTU methods, while minimizing the generation of pseudosequences (Amir et al., 2017). Furthermore, ASVs improve the precision, comprehensiveness, and reproducibility of marker gene analysis, making them a more reliable alternative to

OTUs (Callahan et al., 2019). To annotate each ASV, the classify-sklearn algorithm in QIIME2 was applied, using a pre-trained Naive Bayes classifier (Bokulich et al., 2018). The Silva 138.1 database was utilized for this annotation process. From the ASV annotations and the associated characterization table, we generated a species abundance table, categorizing species at the levels of kingdom, phylum, class, order, family, genus, and species.

A Venn diagram was generated utilizing the "VennDiagram" package in R (Version 2.15.3) to depict the overlapping and unique ASVs between the two groups. Alpha and beta diversity metrics were assessed using QIIME (Version 1.9.1). The Chao1, Observed_features, Shannon, and Pielou_e indices were employed to quantify α-diversity in the gut microbiome. To evaluate β-diversity, Principal Coordinates Analysis (PCoA) and non-metric multidimensional scaling (NMDS) were performed. The linear discriminant analysis effect size (LEfSe) method was applied using LEfSe software (Version 1.0), with an LDA score threshold set to 3.5 (Segata et al., 2011).

13. RNA-seq sequencing

The RNA-seq procedure involved sample preparation, library construction, and quality control. After the library passed the quality assessment, sequencing was performed in PE150 mode using the Illumina NovaSeq6000 platform. Once sequencing data were obtained, bioinformatics analysis was carried out on the BMKCloud platform (www.biocloud.net). The raw data were processed to yield Clean Data, followed by alignment to a reference genome to produce Mapped Data. Subsequently, the data

underwent quality evaluation, structural analysis, differential expression profiling, gene function annotation, and functional enrichment analysis.

14. Intestinal permeability

Intestinal permeability was evaluated by quantifying the absorption of FITC-dextran (Shanghai Yuanye Bio-Technology Co., Ltd, China) after orogastric administration (0.4 g/kg). The diffusion of FITC was monitored using small-animal imaging (IVIS Lumina III, USA). Mice were sacrificed 4 hours post-gavage, and the circulating concentrations of FITC-dextran were determined using established protocols (Cani et al., 2009). Serum diamine oxidase (DAO) activity was measured with a commercial kit (Jiancheng Bioengineering Institute). It is worth noting that in order to avoid the influence of FITC on neferine efficacy, this experiment was carried out independently of other experiments.

15. Molecular docking analysis

The molecular structure of neferine was retrieved from the PubChem Compound Database (Morris et al., 2008). The 3D coordinates of the Keap1 protein (PDB ID: 4IFJ; resolution: 1.80 Å) were obtained from the Protein Data Bank (PDB) (Wang et al., 2017). Molecular docking simulations were conducted using AutoDock Vina v1.1.2 software to assess the binding affinity and interaction modes between neferine and Keap1. Before performing the docking, the protein structure was prepared by removing water molecules, adding hydrogen atoms, and assigning Gasteiger charges through AutoDockTools. Following the docking process, the results were sorted according to

binding affinity scores, and the most optimal complex was chosen for subsequent analysis.

Protein-ligand interactions, such as hydrogen bonds and hydrophobic forces, were visualized and analyzed using PyMOL 2.4.0 software. The analysis identified the critical residues involved in the interaction, offering structural insights into the inhibitory mechanism of neferine and Keap1.

16. Cell proliferation experiment

Cell proliferation was assessed using the CCK-8 assay. Cells were cultured in 96-well plates for 24 hours. After the incubation period, the medium was discarded, and 100 μL of fresh medium containing 10 μL of CCK-8 solution (Biosharp, Hefei, Anhui) was added. After a 2-hour incubation, the optical density at 450 nm was measured using a microplate reader.

17. Simulation of pancreatitis-induced intestinal injury using TNF-α-stimulated AR42J supernatant

To further simulate pancreatitis-induced intestinal injury, we cultured IEC-6 cells with the supernatant from TNF-α-stimulated AR42J cells, using the supernatant from untreated AR42J cells as a control. Specifically, AR42J cells were exposed to TNF-α (50 ng/mL) for 24 hours, washed twice with PBS, and then incubated in fresh culture medium for another 24 hours. The collected supernatant was filtered and added to IEC-6 cell cultures for 24 hours. IEC-6 cell proteins were then extracted for further analysis.

18. Statistics

Data were analyzed using GraphPad Prism 8.0 and are presented as mean ± standard deviation. For datasets following a normal distribution, a t-test was performed to compare two groups, while the Wilcoxon rank sum test was applied for non-normally distributed data. Statistical significance was defined as $P < 0.05$.

Reference

Amir, A., McDonald, D., Navas-Molina, J.A., Kopylova, E., Morton, J.T., Zech Xu, Z., Kightley, E.P., Thompson, L.R., Hyde, E.R., Gonzalez, A., Knight, R., 2017. Deblur Rapidly Resolves Single-Nucleotide Community Sequence Patterns. mSystems 2.

Bokulich, N.A., Kaehler, B.D., Rideout, J.R., Dillon, M., Bolyen, E., Knight, R., Huttley, G.A., Gregory Caporaso, J., 2018. Optimizing taxonomic classification of marker-gene amplicon sequences with QIIME 2's q2-feature-classifier plugin. Microbiome 6, 90.

Bokulich, N.A., Subramanian, S., Faith, J.J., Gevers, D., Gordon, J.I., Knight, R., Mills, D.A., Caporaso, J.G., 2013. Quality-filtering vastly improves diversity estimates from Illumina amplicon sequencing. Nat Methods 10, 57-59.

Callahan, B.J., McMurdie, P.J., Rosen, M.J., Han, A.W., Johnson, A.J., Holmes, S.P., 2016. DADA2: High-resolution sample inference from Illumina amplicon data. Nat Methods 13, 581-583.

Callahan, B.J., Wong, J., Heiner, C., Oh, S., Theriot, C.M., Gulati, A.S., McGill, S.K., Dougherty, M.K., 2019. High-throughput amplicon sequencing of the full-length 16S rRNA gene with single-nucleotide resolution. Nucleic Acids Res 47, e103.

Chiu, C.J., McArdle, A.H., Brown, R., Scott, H.J., Gurd, F.N., 1970. Intestinal mucosal lesion in low-flow states. I. A morphological, hemodynamic, and metabolic reappraisal. Arch Surg 101, 478-483.

Fujimura, K.E., Sitarik, A.R., Havstad, S., Lin, D.L., Levan, S., Fadrosh, D., Panzer, A.R., LaMere, B., Rackaityte, E., Lukacs, N.W., Wegienka, G., Boushey, H.A., Ownby, D.R., Zoratti, E.M., Levin, A.M., Johnson, C.C., Lynch, S.V., 2016. Neonatal gut microbiota associates with childhood multisensitized atopy and T cell differentiation. Nat Med 22, 1187-1191.

Haas, B.J., Gevers, D., Earl, A.M., Feldgarden, M., Ward, D.V., Giannoukos, G., Ciulla, D., Tabbaa, D., Highlander, S.K., Sodergren, E., Methé, B., DeSantis, T.Z., Petrosino, J.F., Knight, R., Birren, B.W., 2011. Chimeric 16S rRNA sequence formation and detection in Sanger and 454-pyrosequenced PCR amplicons. Genome Res 21, 494-504.

Magoč, T., Salzberg, S.L., 2011. FLASH: fast length adjustment of short reads to improve genome assemblies. Bioinformatics 27, 2957-2963.

Morris, G.M., Huey, R., Olson, A.J., 2008. Using AutoDock for ligand-receptor docking. Curr Protoc Bioinformatics Chapter 8, Unit 8.14.

Rognes, T., Flouri, T., Nichols, B., Quince, C., Mahé, F., 2016. VSEARCH: a versatile open source tool for metagenomics. PeerJ 4, e2584.

Segata, N., Izard, J., Waldron, L., Gevers, D., Miropolsky, L., Garrett, W.S., Huttenhower, C., 2011. Metagenomic biomarker discovery and explanation. Genome Biol 12, R60.

Shang, H., Sun, J., Chen, Y.Q., 2016. Clostridium Butyricum CGMCC0313.1 Modulates Lipid Profile, Insulin Resistance and Colon Homeostasis in Obese Mice. PLoS One 11, e0154373.

Shimizu, T., Shiratori, K., Sawada, T., Kobayashi, M., Hayashi, N., Saotome, H., Keith, J.C., 2000. Recombinant human interleukin-11 decreases severity of acute necrotizing pancreatitis in mice. Pancreas 21, 134-140.

Wang, Y., Bryant, S.H., Cheng, T., Wang, J., Gindulyte, A., Shoemaker, B.A., Thiessen, P.A., He, S., Zhang, J., 2017. PubChem BioAssay: 2017 update. Nucleic Acids Res 45, D955-d963.

Zheng, J., Lou, L., Fan, J., Huang, C., Mei, Q., Wu, J., Guo, Y., Lu, Y., Wang, X., Zeng, Y., 2019. Commensal Escherichia coli Aggravates Acute Necrotizing Pancreatitis through Targeting of Intestinal Epithelial Cells. Appl Environ Microbiol 85.

Supplementary figures

Fig. S1. (A) The weight of C57BL/6 mice pre-treated with neferine. (B) Liver function test performed 7 days after treatment, measuring ALT and AST levels. (C) Kidney function test performed 7 days after treatment, measuring creatinine and urea levels. (D) H&E staining of liver, lung, heart, and kidney tissues from mice treated with neferine for 7 days. (E) H&E staining of pancreatic tissue and corresponding histological scores. NE, neferine. Data are presented as mean ± SD; $n = 6$ per group. *$P < 0.05$, ***$P < 0.001$.

Fig. S2. Immunofluorescence staining of F4/80 and CD206 in the pancreas and quantification of pancreatic $F4/80^{+}CD206^{+}$ macrophages. NE, neferine. Data are presented as mean ± SD; $n = 6$ per group. **$P < 0.01$, ***$P < 0.001$.

Fig. S3. (A–C) Immunohistochemical staining of ileal IL-1β (A), IL-6 (B), and TNF-α (C), with corresponding average optical density measurements. (D) Ileal mRNA levels of *Il-1β*, *Il-6*, and *Tnf-α*. AOD, average optical density; NE, neferine. Data are presented as mean ± SD; $n = 6$ per group. **$P < 0.01$, ***$P < 0.001$.

Fig. S4. (A) Immunofluorescence staining of ileal TUNEL and quantification of TUNEL-positive cells. (B) Analysis of relative gray values of ZO-1, occludin, and claudin-1 in the ileum. NE, Neferine; Data were expressed as mean ± SD; n = 6 in each group. *$P < 0.05$, **$P < 0.01$, ***$P < 0.001$.

Fig. S5. Neferine restores intestinal microbiota abundance in SAP. (A) Venn diagram showing the overlap of ASVs identified among the four groups. (B) Distributional differences in gut microbiota profiles assessed using NMDS. (C) Community diversity assessed by Shannon and Pielou_e indices. (D) Taxonomic composition distribution at the genus level in fecal samples from the four groups. (E) Relative abundances of *Lactobacillus*, *Lachnospiraceae_NK4A136_group*, *Alistipes*, *Prevotellaceae_UCG-001*, *Eubacterium_xylanophilum_group*, *Escherichia-Shigella,* and *Enterococcus* at the genus level. Data are expressed as median and quartiles. (F) Fecal levels of isobutyric acid, valeric acid, and isovaleric acid. NE, Neferine; Data are presented as mean ± SD; n = 6 per group. $^*P < 0.05$, $^{**}P < 0.01$, $^{***}P < 0.001$.

Fig. S6. (A) Pancreatic levels of SOD, GSH, and MDA. (B) Ileal levels of SOD, GSH, and MDA. (C) Analysis of relative gray values of ACSL4, xCT, and GPX4 in the pancreas. (D) Analysis of relative gray values of ACSL4, xCT, and GPX4 in the ileum. (E) Immunohistochemical staining of ACSL4, with average optical density quantification in the pancreas and ileum. (F) Analysis of relative gray value of FPN in the pancreas and ileum. AOD, average optical density; NE, Neferine; Data are presented as mean ± SD; n = 6 per group. $^*P < 0.05$, $^{**}P < 0.01$, $^{***}P < 0.001$.

Fig. S7. (A) Immunohistochemical staining of pancreatic IL-1β, IL-6, and TNF-α along with average optical density measurements. (B) Immunohistochemical staining of ileal IL-1βalong with average optical density measurements. (C) Pancreatic mRNA

expression levels of *il-1β*, *il-6*, and *tnf-α*. (D) Ileal mRNA expression levels of *il-1β*, *il-6*, and *tnf-α*. NE, Neferine; Data are presented as mean ± SD; n = 6 per group. **P* < 0.05, ***P* < 0.01, ****P* < 0.001.

Fig. S8. (A) Immunofluorescence staining and quantification of pancreatic and ileal Ly-6G. (B) Immunofluorescence staining and quantification of M1 macrophages ($F4/80^{+}iNOS^{+}$) and M2 macrophages ($F4/80^{+}CD206^{+}$) in the pancreas. NE, Neferine; Data are presented as mean ± SD; n = 6 per group. **P* < 0.05, ***P* < 0.01, ****P* < 0.001.

Fig. S9. (A) Immunofluorescence staining of ileal MUC2 and lysozyme, along with their quantification. (B) Serum levels of DAO and FITC-dextran measured by ELISA. (C). Ileal levels of SOD, GSH, and MDA. (D, E) Relative gray values of ACSL4, xCT, and FPN expression in the pancreas (D) and ileum (E). NE, Neferine; Data are presented as mean ± SD; n = 6 per group. **P* < 0.05, ***P* < 0.01, ****P* < 0.001.

Fig. S10. (A) Analysis of relative gray values of Nrf2, Keap1, HO-1, NQO-1, and nuclear Nrf2 in the pancreas. (B) Analysis of relative gray values of Nrf2, Keap1, HO-1, NQO-1, and nuclear Nrf2 in the ileum. NE, Neferine; Data are presented as mean ± SD; n = 6 per group. **P* < 0.05, ***P* < 0.01, ****P* < 0.001.

Fig. S11. (A) Serum levels of IL-1β, IL-6, TNF-α, and IL-10. (B) Pancreatic mRNA levels of *il-1β*, *il-6*, and *tnf-α*. (C) Ileal mRNA levels of *il-1β*, *il-6*, and *tnf-α*. (D) The

average optical density calculations of TNF-α. NE, Neferine; ML, ML385; Data are presented as mean ± SD; n = 6 per group. *$P < 0.05$, **$P < 0.01$, ***$P < 0.001$.

Fig. S12. (A) Quantification of pancreatic and ileal TUNEL. (B) Immunofluorescence staining and quantification of pancreatic and ileal Ly-6G. (C) Quantification of pancreatic MPO-positive cells. (D) Immunofluorescence staining for M1 macrophages ($F4/80^{+}iNOS^{+}$) and M2 macrophages ($F4/80^{+}CD206^{+}$) in the pancreas and their quantification. (E) Quantification of pancreatic and ileal MUC2. (F) Immunofluorescence staining and quantification of ileal lysozyme (I). NE, Neferine; ML, ML385; Data are presented as mean ± SD; n = 6 per group. **$P < 0.01$, ***$P < 0.001$.

Fig. S13. (A) Quantification of EUB338-positive bacteria in the per field. (B) Western blot analysis of ZO-1, occludin, and claudin-1 protein levels in the ileum, along with relative gray value analysis. (C) Fecal levels of isobutyric acid, valeric acid, and isovaleric acid. (D) Pancreatic and Ileal mRNA levels of *gpx4*, *SLC7A11*, and *fpn*. NE, Neferine; ML, ML385; Data are presented as mean ± SD; n = 6 per group. *$P < 0.05$, **$P < 0.01$, ***$P < 0.001$.

Fig. S14. IEC-6 cells were treated with varying concentrations of neferine (0, 1, 2, 4, 8, 16, 32, or 64 μM) for 24 hours, and cell viability was assessed using the CCK8 assay. NE, Neferine; Data are presented as mean ± SD; n = 6 per group. ***$P < 0.001$.

Fig. S15. (A) Western blot analysis was performed to measure the expression of Nrf2, Keap1, FPN, ACSL4, IL-1β, IL-6, ZO-1, and Occludin in AR42J cells treated with si-Nrf2 and si-FPN. (B) Western blot analysis was performed to measure the expression of nuclear ACSL4, xCT, FPN, IL-1β, and ZO-1 in IEC-6 cells treated with TNF-α-stimulated AR42J supernatant. (C) Western blot analysis was performed to measure the expression of nuclear Nrf2, FPN, ACSL4, IL-1β, IL-6, ZO-1, and Occludin in wild-type and Keap1-mutant IEC-6 cells treated with TNF-α and neferine. NE, Neferine; Data are presented as mean ± SD; n = 6 per group. *$P < 0.05$, **$P < 0.01$, ***$P < 0.001$.

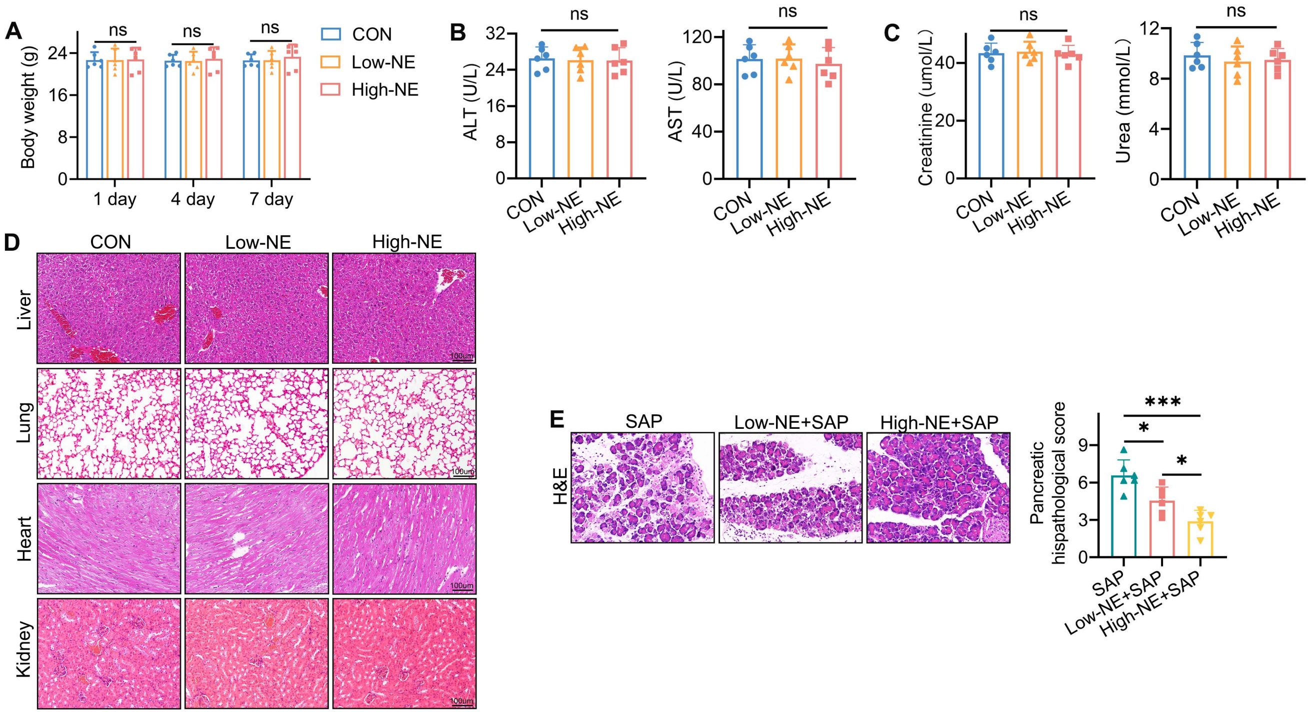

A
Body weight (g)
0
8
16
24
ns
1 day
4 day
7 day
CON
Low-NE
High-NE
B
ALT (U/L)
0
8
16
24
32
AST (U/L)
0
40
80
120
C
Creatinine (umol/L)
0
20
40
Urea (mmol/L)
0
4
8
12
D
CON
Low-NE
High-NE
Liver
Lung
Heart
Kidney
100um
E
SAP
Low-NE+SAP
High-NE+SAP
H&E
Pancreatic hispathological score
0
3
6
9
***
*

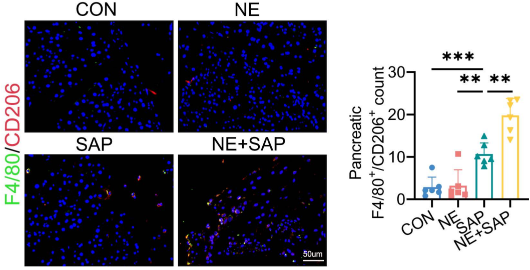
CON
NE
SAP
NE+SAP
F4/80/CD206
50um
***
**
**
Pancreatic
F4/80+/CD206+ count
30
20
10
0
CON
NE
SAP
NE+SAP

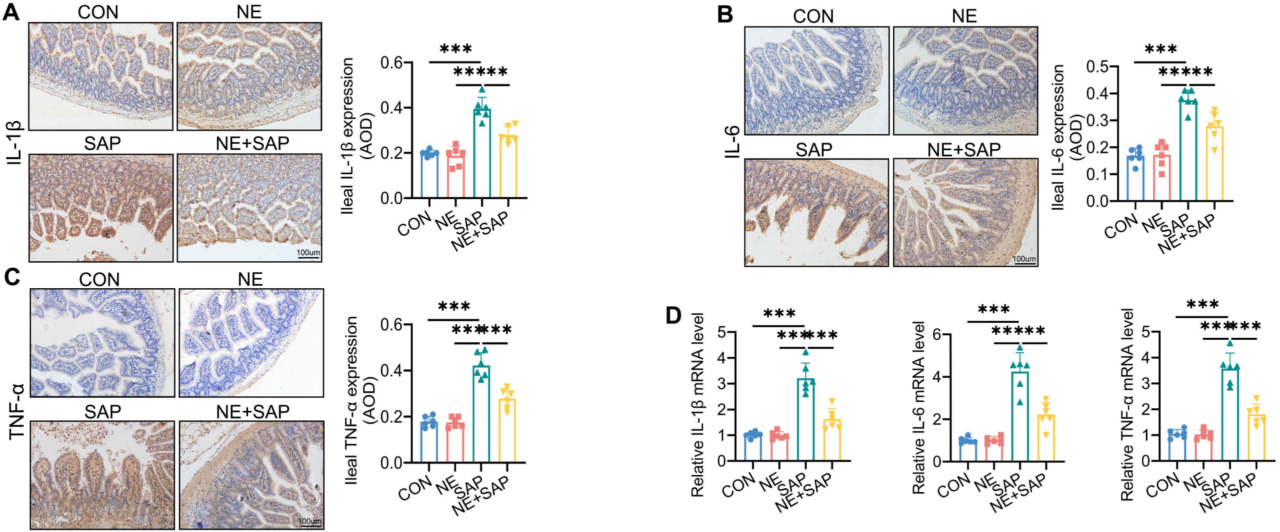
A
CON
NE
SAP
NE+SAP
IL-1β
100um
Ileal IL-1β expression (AOD)
***
***
***
CON
NE
SAP
NE+SAP
B
CON
NE
SAP
NE+SAP
IL-6
100um
Ileal IL-6 expression (AOD)
C
CON
NE
SAP
NE+SAP
TNF-α
100um
Ileal TNF-α expression (AOD)
D
Relative IL-1β mRNA level
Relative IL-6 mRNA level
Relative TNF-α mRNA level

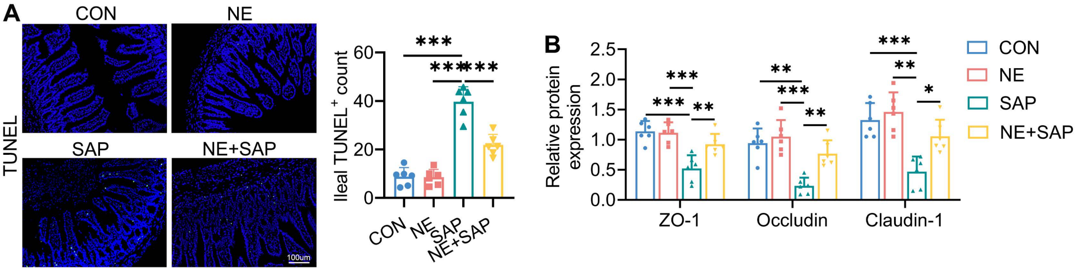
A
CON
NE
SAP
NE+SAP
TUNEL
100um
Ileal TUNEL$^+$ count
60
40
20
0
***
******
CON
NE
SAP
NE+SAP
B
Relative protein expression
2.5
2.0
1.5
1.0
0.5
0.0
ZO-1
Occludin
Claudin-1
***
**
*
CON
NE
SAP
NE+SAP

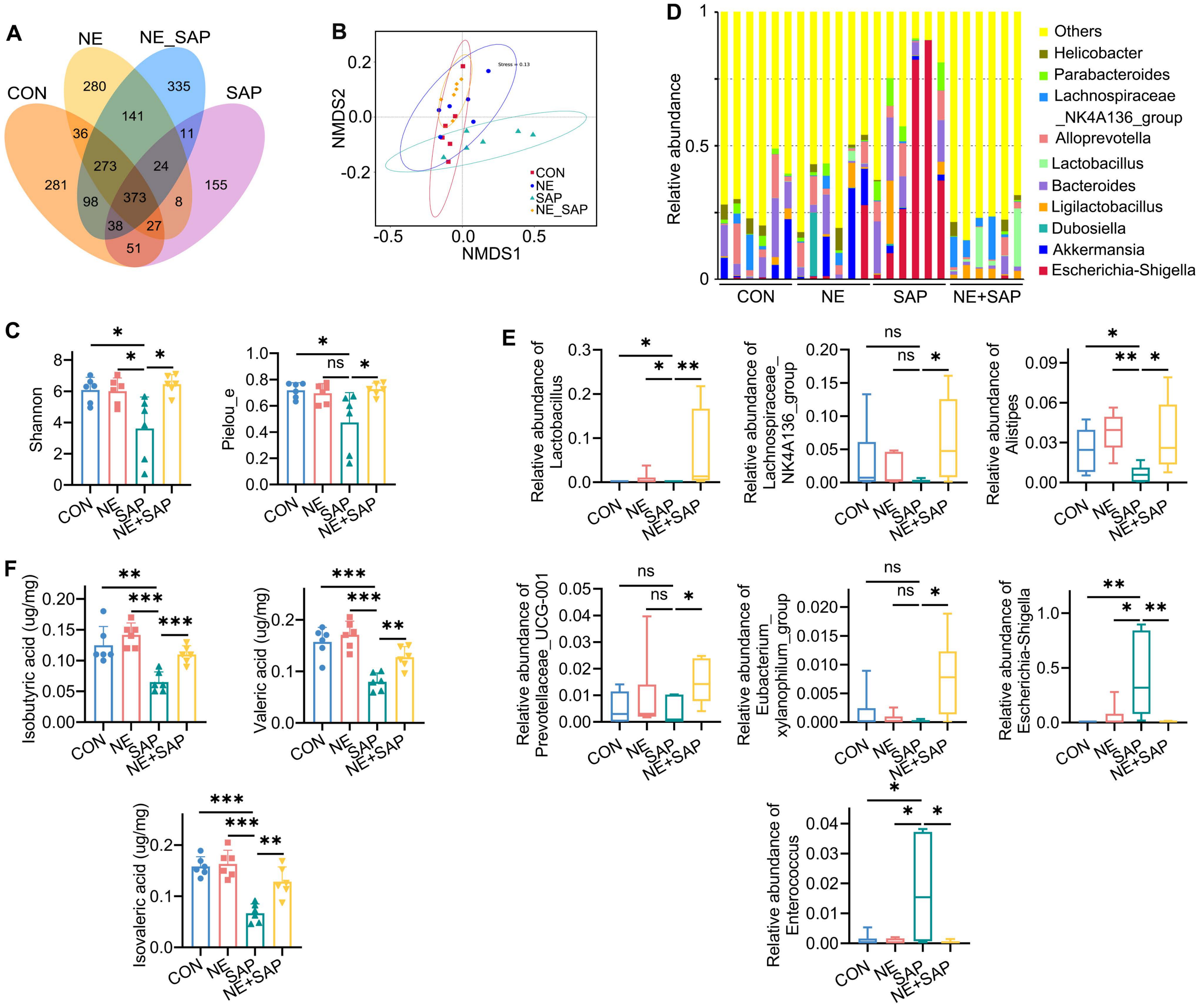
A
CON
NE
NE_SAP
SAP
280
335
141
36
11
273
24
281
155
98
373
8
38
27
51
B
NMDS2
NMDS1
Stress = 0.13
CON
NE
SAP
NE_SAP
D
Relative abundance
Others
Helicobacter
Parabacteroides
Lachnospiraceae_NK4A136_group
Alloprevotella
Lactobacillus
Bacteroides
Ligilactobacillus
Dubosiella
Akkermansia
Escherichia-Shigella
CON
NE
SAP
NE+SAP
C
Shannon
Pielou_e
E
Relative abundance of Lactobacillus
Relative abundance of Lachnospiraceae_NK4A136_group
Relative abundance of Alistipes
Relative abundance of Prevotellaceae_UCG-001
Relative abundance of Eubacterium_xylanophilum_group
Relative abundance of Escherichia-Shigella
Relative abundance of Enterococcus
F
Isobutyric acid (ug/mg)
Valeric acid (ug/mg)
Isovaleric acid (ug/mg)
ns

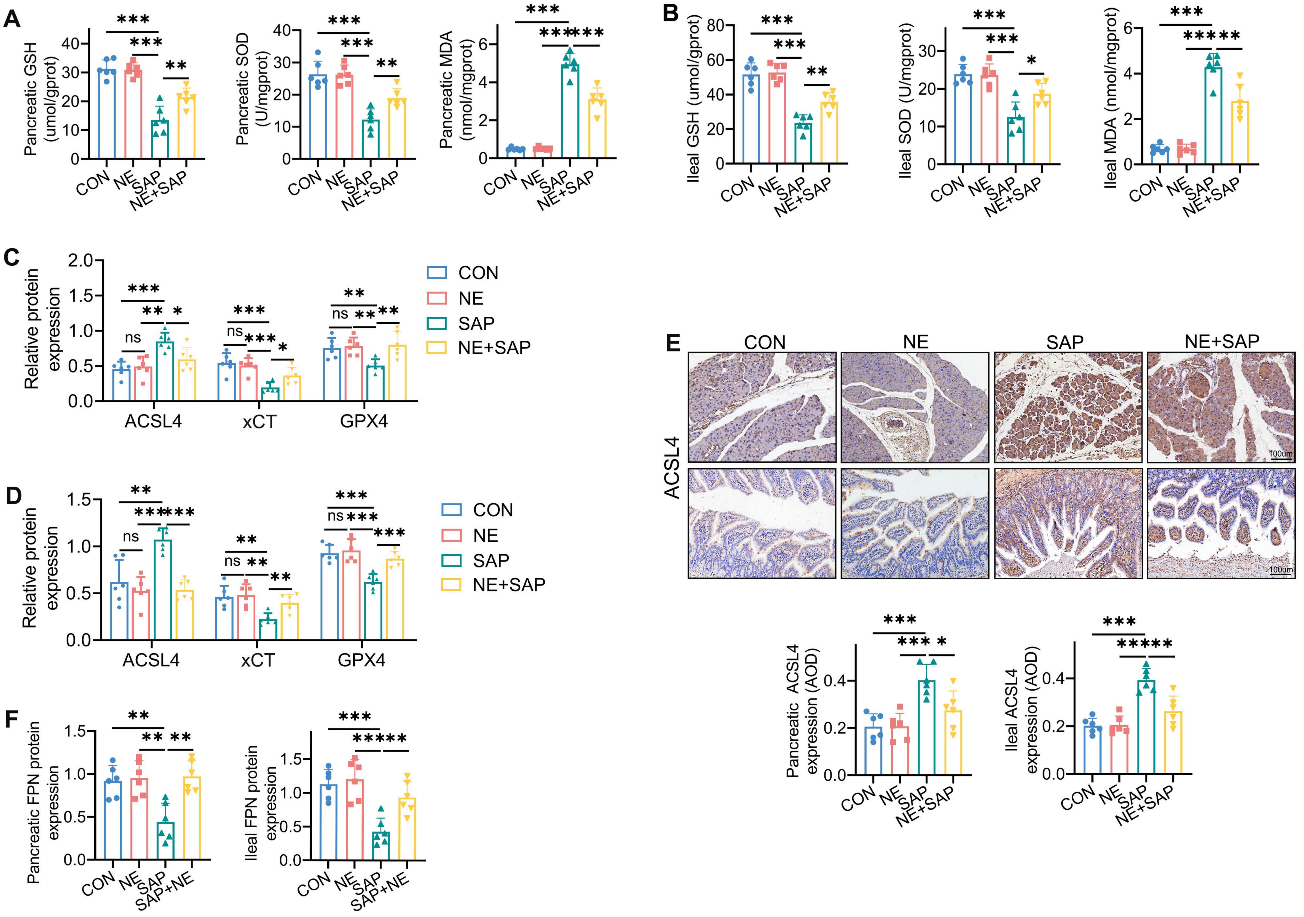
A
Pancreatic GSH (umol/gprot)
Pancreatic SOD (U/mgprot)
Pancreatic MDA (nmol/mgprot)
B
Ileal GSH (umol/gprot)
Ileal SOD (U/mgprot)
Ileal MDA (nmol/mgprot)
CON NE SAP NE+SAP
C
Relative protein expression
ACSL4 xCT GPX4
CON NE SAP NE+SAP
D
Relative protein expression
ACSL4 xCT GPX4
CON NE SAP NE+SAP
E
CON NE SAP NE+SAP
ACSL4
100um
Pancreatic ACSL4 expression (AOD)
Ileal ACSL4 expression (AOD)
F
Pancreatic FPN protein expression
Ileal FPN protein expression
CON NE SAP SAP+NE

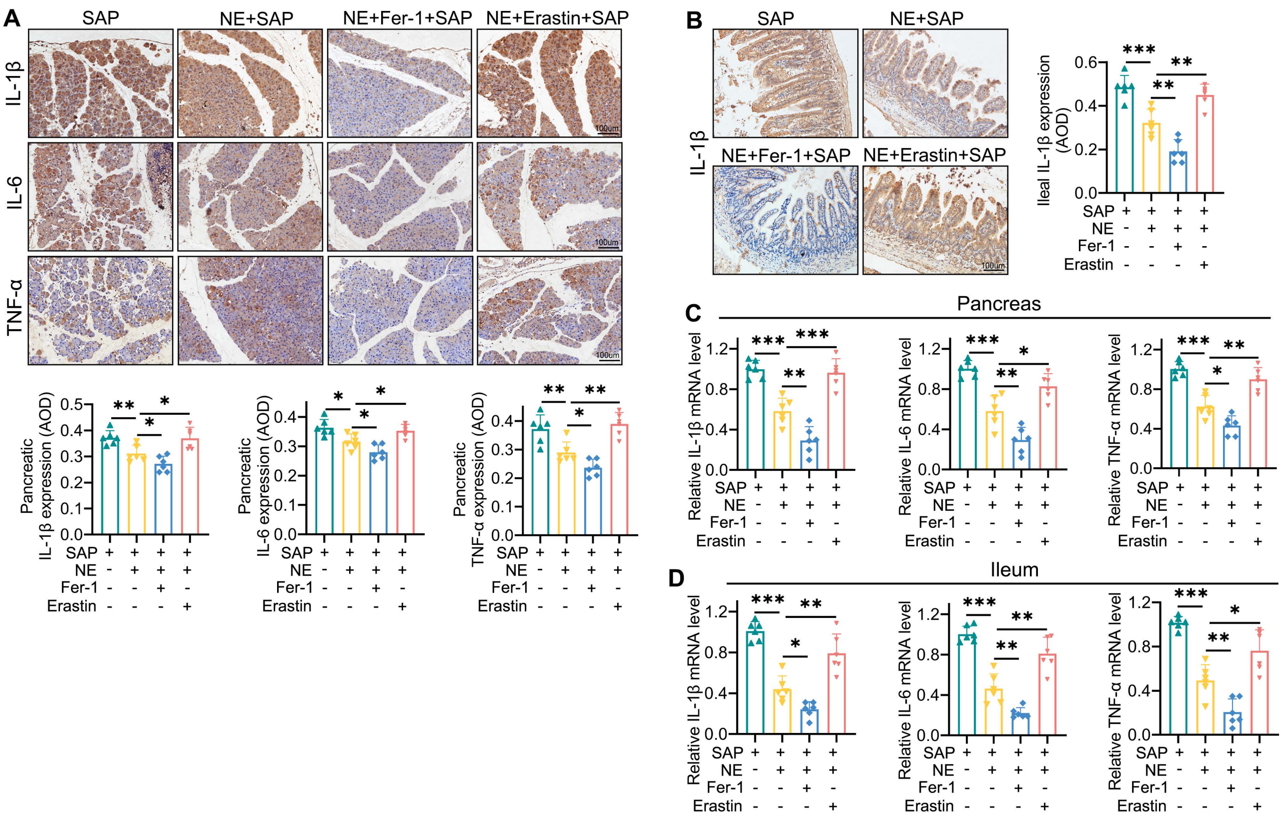

A
SAP
NE+SAP
NE+Fer-1+SAP
NE+Erastin+SAP
IL-1β
IL-6
TNF-α
100um
Pancreatic IL-1β expression (AOD)
Pancreatic IL-6 expression (AOD)
Pancreatic TNF-α expression (AOD)
SAP
NE
Fer-1
Erastin
B
IL-1β
Ileal IL-1β expression (AOD)
C
Pancreas
Relative IL-1β mRNA level
Relative IL-6 mRNA level
Relative TNF-α mRNA level
D
Ileum

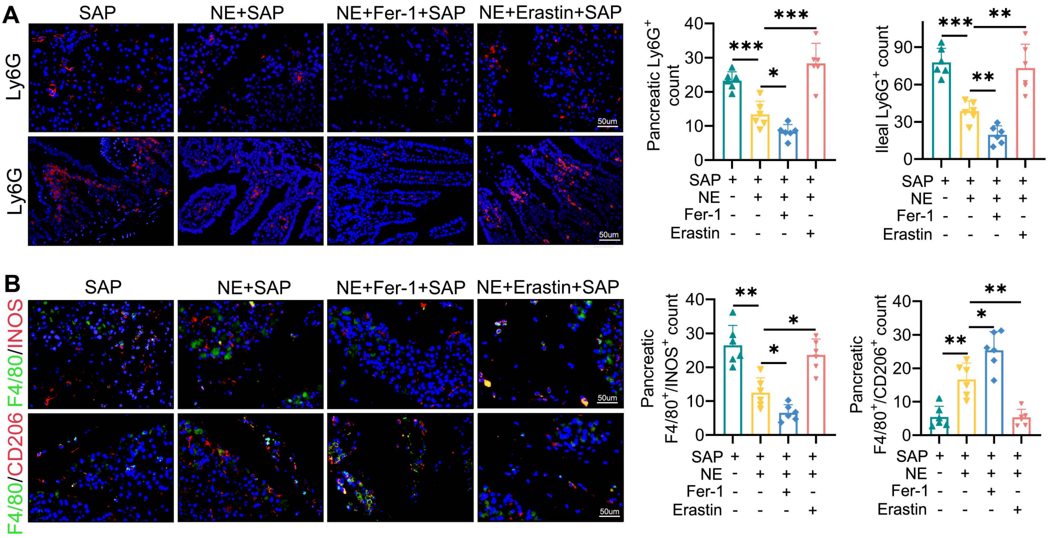
A
SAP
NE+SAP
NE+Fer-1+SAP
NE+Erastin+SAP
Ly6G
Ly6G
50um
50um
Pancreatic Ly6G+ count
Ileal Ly6G+ count
SAP
NE
Fer-1
Erastin
B
SAP
NE+SAP
NE+Fer-1+SAP
NE+Erastin+SAP
F4/80/INOS
F4/80/CD206
50um
50um
Pancreatic F4/80+/INOS+ count
Pancreatic F4/80+/CD206+ count
SAP
NE
Fer-1
Erastin

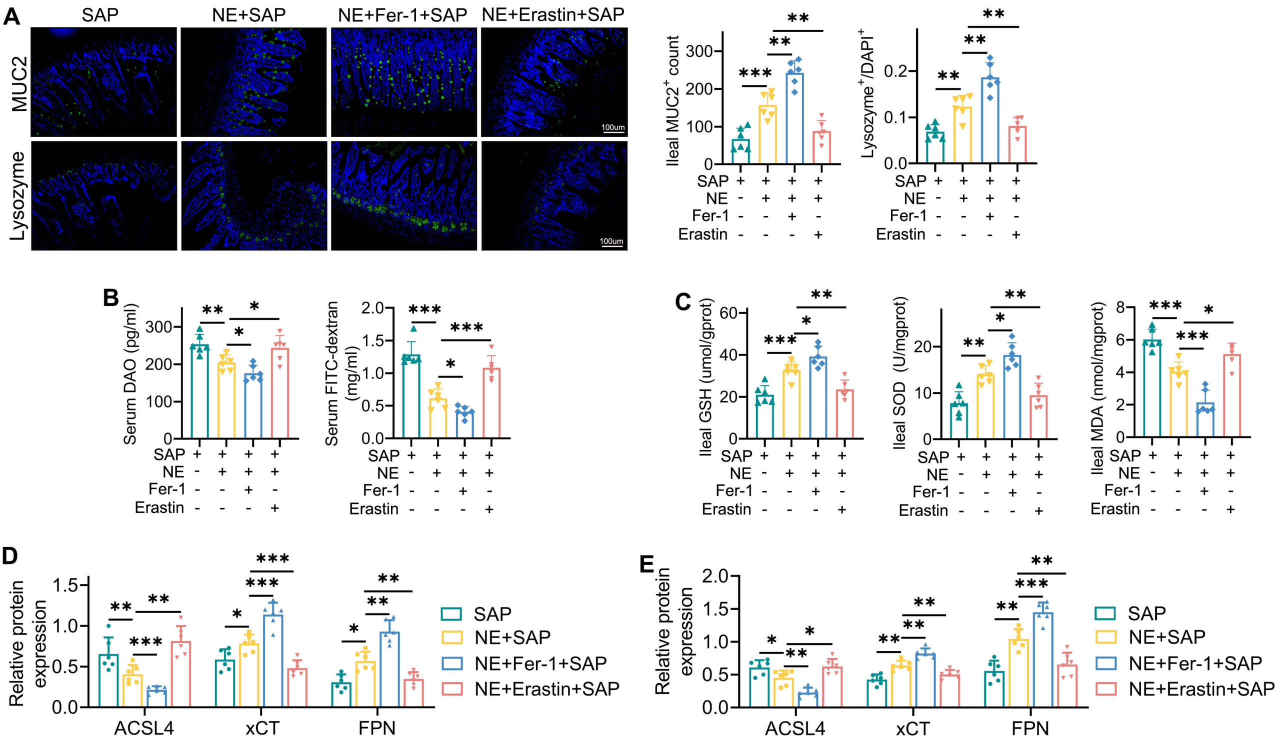
A
SAP
NE+SAP
NE+Fer-1+SAP
NE+Erastin+SAP
MUC2
Lysozyme
100um
100um
Ileal MUC2$^+$ count
Lysozyme$^+$/DAPI$^+$
SAP + + + +
NE - + + +
Fer-1 - - + -
Erastin - - - +
B
Serum DAO (pg/ml)
Serum FITC-dextran (mg/ml)
C
Ileal GSH (umol/gprot)
Ileal SOD (U/mgprot)
Ileal MDA (nmol/mgprot)
D
E
Relative protein expression
ACSL4
xCT
FPN
SAP
NE+SAP
NE+Fer-1+SAP
NE+Erastin+SAP

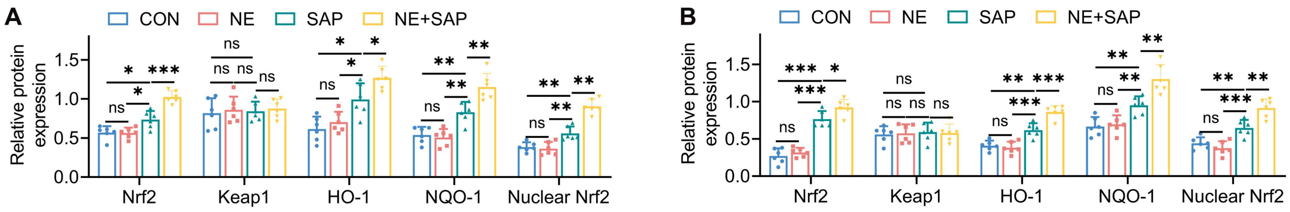
A
B
CON
NE
SAP
NE+SAP
Relative protein expression
Nrf2
Keap1
HO-1
NQO-1
Nuclear Nrf2
ns
0.0
0.5
1.0
1.5

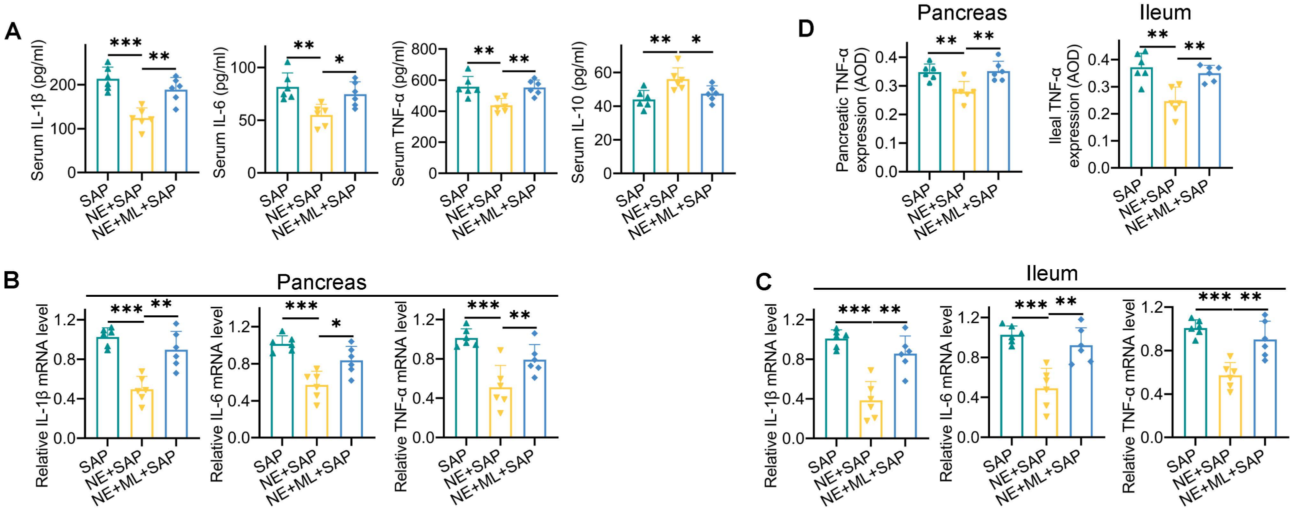

A
Serum IL-1β (pg/ml)
Serum IL-6 (pg/ml)
Serum TNF-α (pg/ml)
Serum IL-10 (pg/ml)
SAP
NE+SAP
NE+ML+SAP
B
Pancreas
Relative IL-1β mRNA level
Relative IL-6 mRNA level
Relative TNF-α mRNA level
C
Ileum
D
Pancreas
Ileum
Pancreatic TNF-α expression (AOD)
Ileal TNF-α expression (AOD)

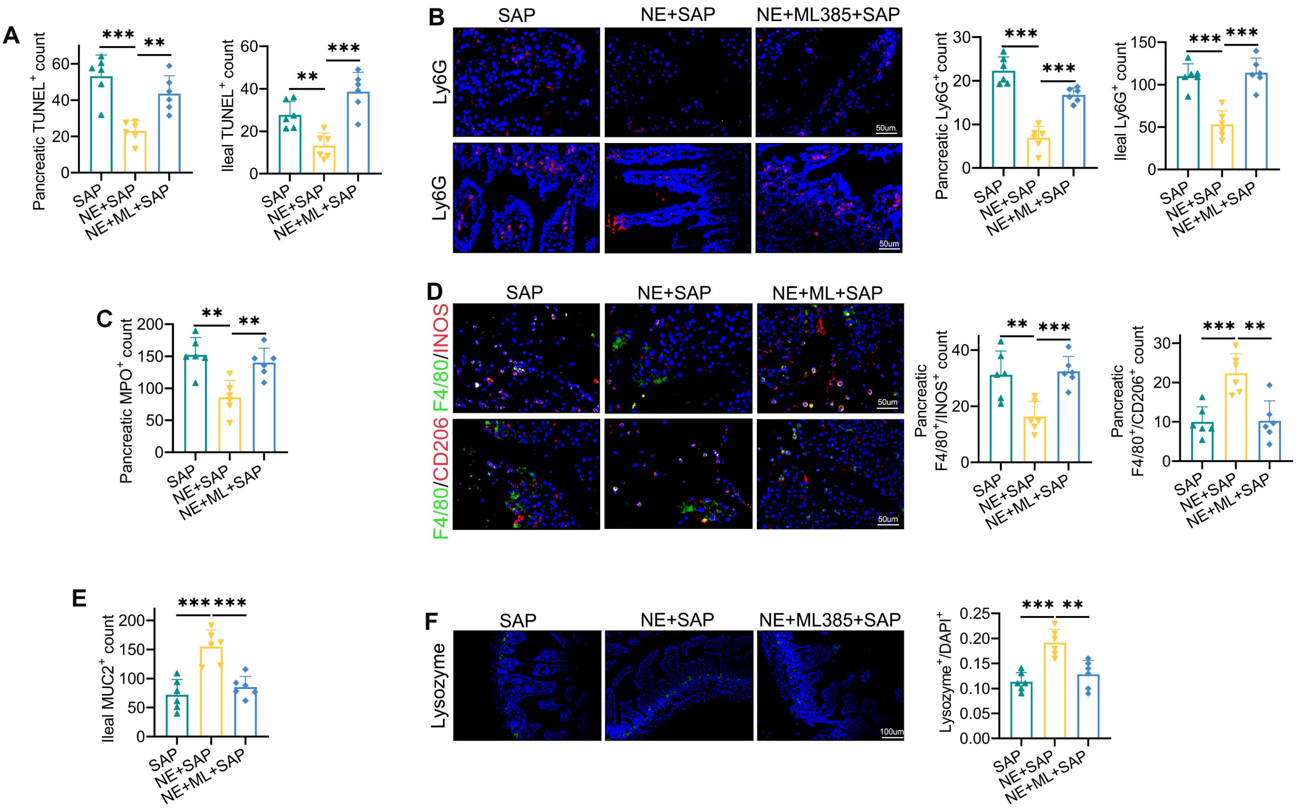

A
Pancreatic TUNEL+ count
Ileal TUNEL+ count
SAP
NE+SAP
NE+ML+SAP
B
SAP
NE+SAP
NE+ML385+SAP
Ly6G
50um
Pancreatic Ly6G+ count
Ileal Ly6G+ count
C
Pancreatic MPO+ count
D
NE+ML+SAP
F4/80/INOS
F4/80/CD206
Pancreatic F4/80+/INOS+ count
Pancreatic F4/80+/CD206+ count
E
Ileal MUC2+ count
F
Lysozyme
100um
Lysozyme+/DAPI+

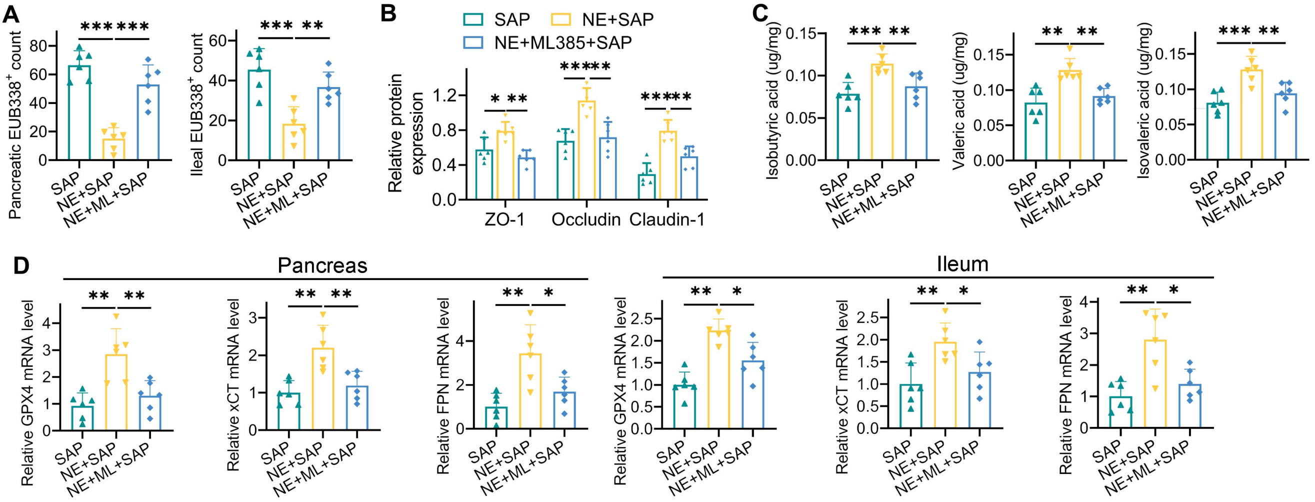
A
Pancreatic EUB338+ count
Ileal EUB338+ count
SAP
NE+SAP
NE+ML+SAP
B
SAP
NE+SAP
NE+ML385+SAP
Relative protein expression
ZO-1
Occludin
Claudin-1
C
Isobutyric acid (ug/mg)
Valeric acid (ug/mg)
Isovaleric acid (ug/mg)
D
Pancreas
Ileum
Relative GPX4 mRNA level
Relative xCT mRNA level
Relative FPN mRNA level

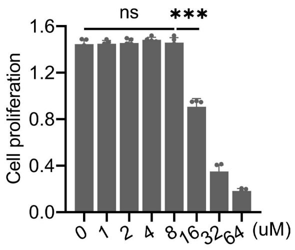

ns
***
1.6
1.2
0.8
0.4
0.0
Cell proliferation
0
1
2
4
8
16
32
64
(uM)

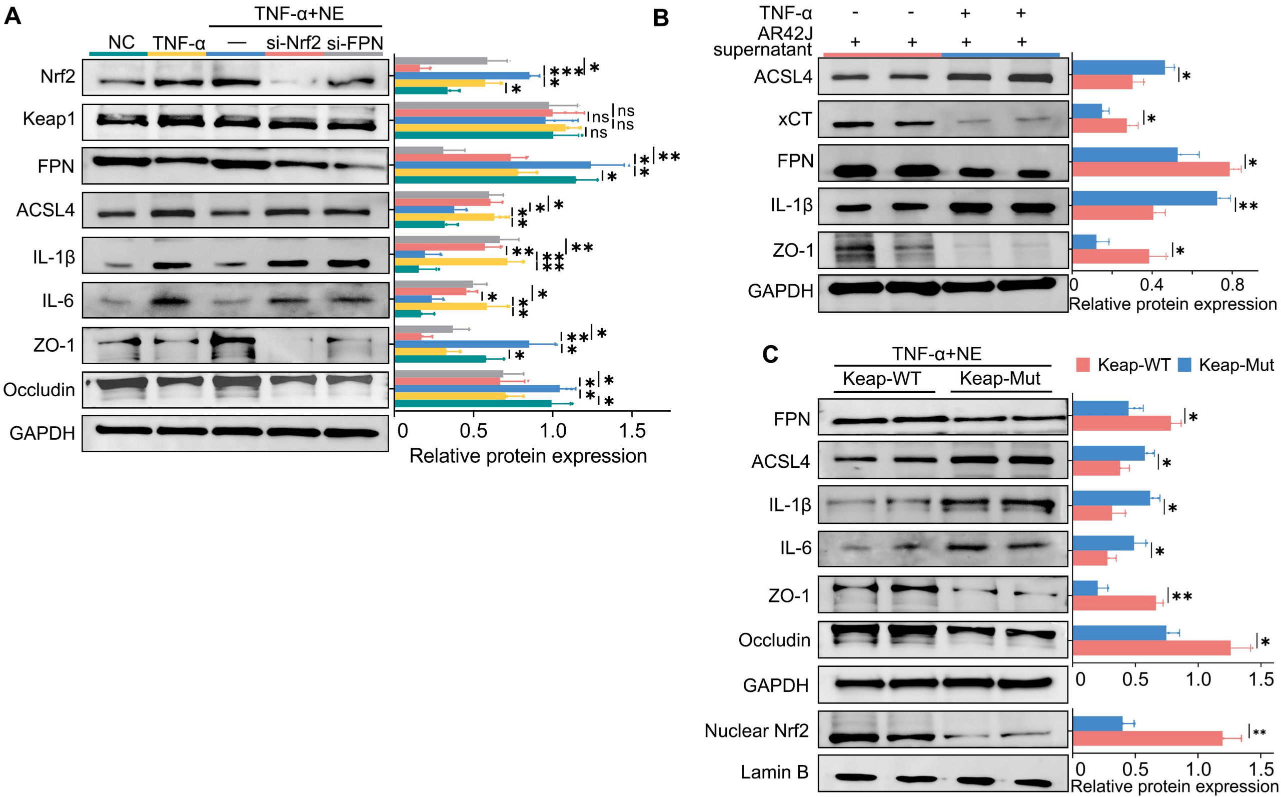

A
TNF-α+NE
NC
TNF-α
—
si-Nrf2
si-FPN
Nrf2
Keap1
FPN
ACSL4
IL-1β
IL-6
ZO-1
Occludin
GAPDH
0
0.5
1.0
1.5
Relative protein expression
B
TNF-α
AR42J supernatant
- - + +
+ + + +
ACSL4
xCT
FPN
IL-1β
ZO-1
GAPDH
0
0.4
0.8
Relative protein expression
C
TNF-α+NE
Keap-WT
Keap-Mut
FPN
ACSL4
IL-1β
IL-6
ZO-1
Occludin
GAPDH
Nuclear Nrf2
Lamin B
0
0.5
1.0
1.5
Relative protein expression
